# Signature Search Polestar: a comprehensive drug repurposing method evaluation assistant for customized oncogenic signature

**DOI:** 10.1093/bioinformatics/btae536

**Published:** 2024-08-30

**Authors:** Jinbo Zhang, Shunling Yuan, Wen Cao, Xianrui Jiang, Cheng Yang, Chenchao Jiang, Runhui Liu, Wei Yang, Saisai Tian

**Affiliations:** Department of Phytochemistry, School of Pharmacy, Second Military Medical University, Shanghai 200433, China; Department of Pharmacy, Tianjin Rehabilitation Center of Joint Logistics Support Force, Tianjin 300110, China; Department of Phytochemistry, School of Pharmacy, Second Military Medical University, Shanghai 200433, China; Department of Phytochemistry, School of Pharmacy, Second Military Medical University, Shanghai 200433, China; Changhai Hospital, Second Military Medical University, Shanghai 200433, China; Department of Clinical Nutrition, Tianjin Rehabilitation Center of Joint Logistics Support Force, Tianjin 300110, China; Department of Pharmacy, Tianjin Rehabilitation Center of Joint Logistics Support Force, Tianjin 300110, China; Department of Phytochemistry, School of Pharmacy, Second Military Medical University, Shanghai 200433, China; Department of Pharmacy, Tianjin Rehabilitation Center of Joint Logistics Support Force, Tianjin 300110, China; Department of Phytochemistry, School of Pharmacy, Second Military Medical University, Shanghai 200433, China; Key Laboratory of Molecular Pharmacology and Drug Evaluation (Yantai University), Ministry of Education, Yantai University, Yantai 264005, China

## Abstract

**Summary:**

The burgeoning high-throughput technologies have led to a significant surge in the scale of pharmacotranscriptomic datasets, especially for oncology. Signature search methods (SSMs), utilizing oncogenic signatures formed by differentially expressed genes through sequencing, have been instrumental in anti-cancer drug screening and identifying mechanisms of action without relying on prior knowledge. However, various studies have found that different SSMs exhibit varying performance across pharmacotranscriptomic datasets. In addition, the size of the oncogenic signature can also significantly impact the result of drug repurposing. Therefore, finding the optimal SSMs and customized oncogenic signature for a specific disease remains a challenge. To address this, we introduce Signature Search Polestar (SSP), a webserver integrating the largest pharmacotranscriptomic datasets of anti-cancer drugs from LINCS L1000 with five state-of-the-art SSMs (XSum, CMap, GSEA, ZhangScore, XCos). SSP provides three main modules: Benchmark, Robustness, and Application. Benchmark uses two indices, Area Under the Curve and Enrichment Score, based on drug annotations to evaluate SSMs at different oncogenic signature sizes. Robustness, applicable when drug annotations are insufficient, uses a performance score based on drug self-retrieval for evaluation. Application provides three screening strategies, single method, SS_all, and SS_cross, allowing users to freely utilize optimal SSMs with tailored oncogenic signature for drug repurposing.

**Availability and implementation:**

SSP is free at https://web.biotcm.net/SSP/. The current version of SSP is archived in https://doi.org/10.6084/m9.figshare.26524741.v1, allowing users to directly use or customize their own SSP webserver.

## 1 Introduction

Drug repurposing emerges as an alternative and cost-effective strategy for developing new cancer therapies, leveraging old drugs for new therapeutic purposes (Zhang *et al.* 2020). Meanwhile, the exponential increase in pharmacotranscriptomics, fueled by high-throughput technologies like next-generation sequencing and microarrays, is valuable for understanding drug responses but presents challenges in effective management and analysis. The signature search method (SSM), widely used in various omics, can screen potential drugs and identify their mechanisms of action without relying on prior knowledge (Duan *et al.* 2020). In classic anti-cancer drug repurposing research, animal/cellular models or cancer patient cohorts are constructed through experiments or clinical recruitment, then followed by sequencing to obtain a list of differentially expressed genes (DEGs) with leading fold change. Subsequently, an oncogenic signature is proposed based on the top number of up-regulated and down-regulated genes (TopN) in DEGs. Finally, the SSM is used to score the oncogenic signature against existing drugs in the pharmacotranscriptomic dataset, resulting in drug–cancer response metrics. Drugs with negative scores are considered potentially therapeutic agents for cancers (Lv *et al.* 2018, He *et al.* 2023). The LINCS L1000 is the world’s largest pharmacotranscriptomic database recording the effects of >10 000 drugs on gene expression across various tumor cell types (Subramanian *et al.* 2017). Connectivity Map, one of the most successful computational platforms relying on LINCS L1000, enables researchers to explore potential new uses for existing drugs, thereby accelerating the process of anti-cancer drug discovery and development.

However, determining the optimal SSM and identifying the appropriate TopN of oncogenic signature remain challenges. Each SSM and different size of the oncogenic signature may vary against a specific pharmacotranscriptomic dataset, necessitating careful evaluation (Lin *et al.* 2020, Samart *et al.* 2021). Furthermore, the application of multiple SSMs, grounded on drug annotations specific to a particular cancer, often yields more accurate screening results, thereby significantly reducing the cost of wet-lab experiments (Väremo *et al.* 2013, Yang *et al.* 2022). This highlights the need for customized computational tools.

To address the existing gap, we have developed Signature Search Polestar (SSP), a webserver that integrates the largest uniform pharmacotranscriptomic datasets from LINCS L1000 with five state-of-the-art SSMs. SSP allows users to evaluate these SSMs using drug–cancer response metrics with drug annotations and suggests the optimal method and appropriate TopN to construct a customized oncogenic signature. In addition, SSP offers three effective approaches to query drugs for new repurposing based on the specific oncogenic signature.

## 2 Feature

The SSP has built-in pharmacotranscriptomic datasets from LINCS L1000 PHASE I (GSE92742) for drug repurposing, classified by cell line and concentration. In our processing, we retained pharmacotranscriptomic datasets that satisfied two specific criteria: (i) The source cell lines were part of the Core Cell Line Panel, and (ii) The datasets comprised sequencing data for >100 drugs, emphasizing the potential for drug repurposing. Ultimately, we included pharmacotranscriptomic datasets encompassing over 100 drugs, resulting in a refined collection of 49 pharmacotranscriptomic datasets in nine tumor cell lines.

Furthermore, given that these pharmacotranscriptomic datasets predominantly originate from tumor cell lines, SSP also integrates preliminary drug annotations sourced from the Genomics of Drug Sensitivity in Cancer database (GDSC) and FDA-approved drug repurposing information from Drug Repurposing Hub (Yang *et al.* 2013, Corsello *et al.* 2017).

**Figure 1.**
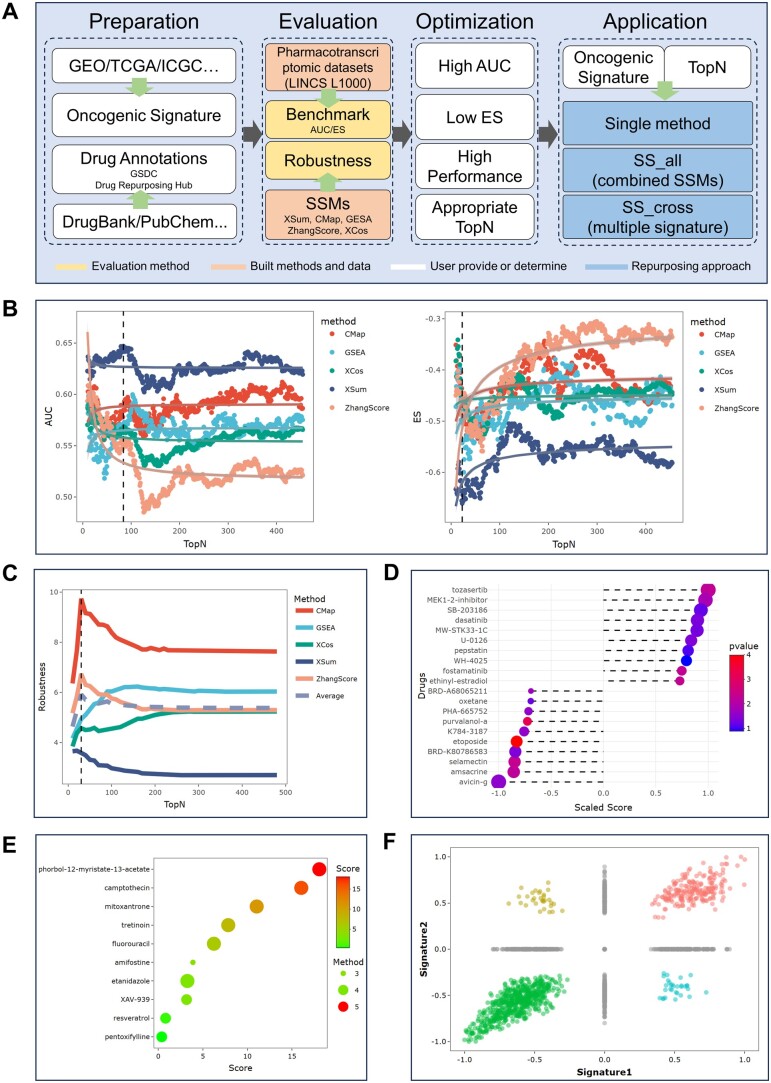
The workflow and result demonstration of SSP. (A) The architecture of the SSP webserver. (B) The result of AUC (left) and ES (right) in the Benchmark module. The AUC assesses the method’s ability to distinguish between effective and ineffective drugs, with a higher score indicating better performance. The ES evaluates if effective drugs are enriched at the top of the drug list, sorted in descending order based on scores, with a lower score indicating better performance. (C) The result of the Robustness module. A higher score indicates better performance. (D) The result of the Single method in the Application module displays the top five drugs with the highest positive scores and the top five drugs with the highest negative scores. Positive values indicate drugs with a potentially agonistic effect on the disease, while negative values indicate drugs with a potentially therapeutic effect. (E) The result of the SS_all method in the Application module, displaying the top 10 drugs with significant scores in the same direction. The top drug is more likely to be promising as it is also the highest-ranked drug in most SSMs. The dot size represents the number of SSMs where the drug is retrieved. (F) The result of SS_cross in the Application module, where all drugs are plotted in a scatter plot based on their scores on the *x*–*y* plane. Different quadrants represent different effects (agonistic >0 or therapeutic <0) of the drug against the oncogenic signature. Drugs in the lower left corner are more promising.

The workflow of SSP, as shown in Fig. 1A, mainly consists of the following primary modules: Benchmark, Robustness, and Application. The Benchmark module conducts evaluations leveraging external drug annotations, whereas the Robustness module evaluates through drug self-retrieval (without annotation). These two modules provide insights into the optimal SSM and TopN for a given oncogenic signature. These insights and the oncogenic signature are then applied in the Application module for drug repurposing.

Complementing these core modules, SSP also features four auxiliary modules: Annotation, Job Center, Converter, and Info module. These auxiliary modules support the aforementioned primary modules by offering data preprocessing and result retrieval functionalities. Detailed methodology, result interpretation, and an example study case using SSP are provided in the Supplementary Document.

### 2.1 Benchmark module

In the Benchmark module, users are required to upload an initial oncogenic signature (representing cancer) and at least one drug annotation file and to select a pharmacotranscriptomic dataset of interest. Subsequently, the oncogenic signature undergoes iterative tailored filtration for assessing the performance of SSMs. Specifically, the genes in the oncogenic signature are ordered based on leading fold change, then an equivalent number (TopN) of significantly up-regulated and down-regulated genes are selected to form a new oncogenic signature. This approach guarantees a comprehensive and balanced representation of the varied gene expression alterations associated with the disease state. Then, five state-of-the-art SSMs, XSum (Cheng *et al.* 2014), CMap (Lamb *et al.* 2006), GSEA (Subramanian *et al.* 2005), ZhangScore (Zhang and Gant 2008), and XCos (Cheng *et al.* 2013) are called to calculate scores. We compute the scores for these SSMs across a range of TopN values to obtain drug–cancer response metrics. In general, a score <0 indicates that the drug is potentially agonistic to the cancer, while a score <0 indicates that the drug is potentially therapeutic to the cancer.

Next, based on the drug annotation data, two benchmarking indices, namely area under the curve (AUC) and enrichment score (ES), are generated for evaluating the performance of SSMs at different TopN. The methodologies of AUC and ES are derived from commonly used metrics for evaluating drug efficacy (Yang *et al.* 2022). Within the Benchmark module, these benchmarking standards can be adeptly used to utilize the scores based on pharmacotranscriptomic datasets and annotation information, thereby facilitating an accurate assessment of the precision of SSMs. The AUC uses scores of drugs annotated with “Effective” and “Ineffective,” and aims to assess whether the SSM can effectively discriminate between these categories, with a higher score signifying a more reliable method. This metric applies to drug annotations derived from large-scale experimental screenings, such as determining drug efficacy based on IC_50_ values. The ES aims to evaluate whether the annotated drugs can be enriched at the top of the descending ordered list of all drugs based on scores from SSM, with a negative score indicating a better method. This metric is suitable for drug annotations based on clinical practice, where a minority of drugs are known to be effective, and the efficacy of the majority remains uncertain.

When combined, these metrics offer a comprehensive assessment of the predictive performance of SSMs under different TopN, encompassing both clinical and experimental perspectives. As shown in Fig. 1B, the evaluation results are presented in scatter plots, with the *x*-axis representing the number of extracted DEGs (TopN) in the oncogenic signature, and the *y*-axis representing the evaluation score (either AUC or ES). A vertical line has been added to the plot to denote the most significant value (with AUC representing the highest value and ES the lowest). Concurrently, the table has been adjusted to highlight the row containing the most significant value in the first row. Users can select SSMs and TopN candidates that demonstrate strong performance across both metrics for further research.

### 2.2 Robustness module

The purpose of the Robustness module is to assess the performance of SSMs in scenarios where drug annotations are limited, such as for subtypes of cancer or rare cancers. This approach resembles a drug self-retrieval process, harnessing the “drug signatures” generated from the pharmacotranscriptomic dataset to compute drug-drug similarity metrics. These similarity metrics are then evaluated using the performance score developed by our team in previous publications (Tian *et al.* 2023b). Robustness, akin to traditional SSM comparison, yields pre-computed outcomes and varies across different pharmacotranscriptomic datasets. As shown in Fig. 1C, the evaluation results are presented in scatter plots, with the *x*-axis representing the number of extracted DEGs (TopN) in the oncogenic signature, the *y*-axis representing the performance score, and a higher score indicating a better method.

### 2.3 Application module (query drugs)

The Benchmark module and Robustness module allow users to determine the optimal SSMs and number of TopN genes for the best performance on a specific oncogenic signature, and the Application module provides three powerful approaches for drug repurposing.

The “single method” is a classic method. One oncogenic signature and one SSM are required to retrieve drugs. As presented in Fig. 1D, drugs with the potential to reverse cancer progression may exhibit a negative score. The “SS_all” method and “SS_cross” method were designed to find promising drugs with consensus under multiple SSMs or oncogenic signatures (Tian *et al.* 2023b). In the SS_all, the user is required to select ***all*** the SSMs with higher performance in Benchmark or Robustness. Then, drug–cancer response metrics are generated by all optimal SSMs against an oncogenic signature. Drug rankings are integrated by the robust rank aggregation (Kolde *et al.* 2012) for metrics that align in direction, and each drug is assigned an overall score of −log(*P*-value) derived from the aggregation. Hence, a drug that achieves top ranks across multiple SSMs garners a higher aggregate score and indicates greater potential to be promising. (Fig. 1E). In SS_cross, the user is required to prepare two distinct oncogenic signatures and then drug–cancer response metrics are generated by these oncogenic signatures with one SSM. The purpose of SS_cross is to identify drugs that exhibit strong responses ***across*** two distinct oncogenic signatures. This aligns with the current practice in omics data mining (Liu *et al.* 2024), where findings typically require validation across multiple datasets. These drugs are then divided into four quadrants based on the sign of these metrics (Chen *et al.* 2021), representing potentially agonistic response (>0) or therapeutic response (<0) (Fig. 1F). The drugs that exhibit therapeutic responses in both oncogenic signatures show promise for repurposing, particularly those located in the lower-left corner of the figure.

### 2.4 Auxiliary modules

In addition, SSP offers additional modules to enhance the user experience.

Job Center module: To accommodate the substantial computational demands of the SSP website, a unique job ID is assigned upon successful submission. Users can access the results by entering the job ID in the Job Center module.

Annotation module: SSP provides the preliminary drug annotation to facilitate the user’s manual drug annotation. For AUC, this module integrates annotation data for 286 drugs in 30 cancers sourced from the GDSC database. A threshold of 10 μM is applied, classifying drugs below this value as effective and those above as ineffective. To address IC_50_ redundancy, the median IC_50_ value for duplicate drugs is utilized to represent their activity. Every cancer type is covered with at least 271 drugs (Supplementary Table S1). For ES, the module includes indication-based annotation data for 163 FDA-approved drugs across 15 cancer types, curated manually from the Drug Repurposing Hub database, with a minimum of five drugs annotated per cancer type (Supplementary Table S2). Users can directly download annotation files for the Benchmark module corresponding to ES and AUC.

Converter module: To address the diversity of gene and drug identifiers, SSP offers a Converter module that facilitates the conversion of identifiers to a compatible format in Benchmark. The Converter module exclusively retrieves genes and drugs present within the pharmacotranscriptomic datasets.

Info module: SSP features an Info module offering help pages, demo files, scripts, and curated LINCS L1000 pharmacotranscriptomic datasets organized by concentration and cell line.

## 3 Dependencies and libraries

SSP is a GUI web application based on the Shiny package in R 4.4.0. SQLite is used for storing computational results. The *plotly* and *DT* packages are used for interactive figures and charts, and the *parallel* and *memoise* packages are used to speed up and cache step-by-step computational results. SSP is deployed in a Linux server with 96 CPUs and 768G of memory.

## 4 Conclusion

The growing interest in pharmacotranscriptomics presents challenges in management and analysis. The SSM is proven to be a practical way to screen promising drugs for new use. However, determining the optimal SSM and identifying tailored sizes for specific oncogenic signature remain challenges. SSP is a user-friendly webserver with core functionality that relies on user-provided oncogenic signature and known drug annotations. It utilizes LINCS L1000 pharmacotranscriptomic datasets and five advanced SSMs to score each drug using specific oncogenic signatures with different numbers of differentially expressed genes. These scores are then aggregated to determine the optimal SSM and an oncogenic signature with tailored TopN. Once these optimal conditions are determined, users can utilize the refined criteria for better drug repurposing. SSP serves as a cornerstone for research within the burgeoning field of pharmacotranscriptomics in oncology.

Although the current scope of SSP is primarily focused on the field of cancer, it is believed that with the advancements in high-throughput sequencing and the surge in omics data, the application of SSP can be extended to a wider spectrum of pharmacotranscriptomic databases, such as those in natural product (Tian *et al.* 2023b). Moreover, the methodologies used within SSP may extend beyond oncogenic signature and warrant comprehensive exploration. Future development of SSP will consider the integration of diverse signatures, including those derived from molecularly driven and drug-induced experiments (Tian *et al.* 2023a).

## Supplementary Material

btae536_Supplementary_Data

## Data Availability

The data underlying this article are available in Figshare at https://doi.org/10.6084/m9.figshare.26524741.v1. The datasets were derived from sources in the public domain: Pharmacotranscriptomic datasets are curated from LINCS L1000 PHASE I (https://www.ncbi.nlm.nih.gov/geo/query/acc.cgi?acc=GSE92742), information on drugs are curated from Genomics of Drug Sensitivity in Cancer (https://www.cancerrxgene.org/), and Drug Repurposing Hub (https://www.broadinstitute.org/drug-repurposing-hub).

## References

[btae536-B1] Chen S , Liu X, Peng C et al The phytochemical hyperforin triggers thermogenesis in adipose tissue via a Dlat-AMPK signaling axis to curb obesity. Cell Metab 2021;33:565–80.e7. 33657393 10.1016/j.cmet.2021.02.007

[btae536-B2] Cheng J , Xie Q, Kumar V et al Evaluation of analytical methods for connectivity map data. Pac Symp Biocomput 2013;22:5–16. 23424107

[btae536-B3] Cheng J , Yang L, Kumar V et al Systematic evaluation of connectivity map for disease indications. Genome Med 2014;6:540. 25606058 10.1186/s13073-014-0095-1 PMC4278345

[btae536-B4] Corsello SM , Bittker JA, Liu Z et al The drug repurposing hub: a next-generation drug library and information resource. Nat Med 2017;23:405–8. 28388612 10.1038/nm.4306 PMC5568558

[btae536-B5] Duan Y , Evans DS, Miller RA et al signatureSearch: environment for gene expression signature searching and functional interpretation. Nucleic Acids Res 2020;48:e124. 33068417 10.1093/nar/gkaa878 PMC7708038

[btae536-B6] He H , Duo H, Hao Y et al Computational drug repurposing by exploiting large-scale gene expression data: strategy, methods and applications. Comput Biol Med 2023;155:106671. 36805225 10.1016/j.compbiomed.2023.106671

[btae536-B7] Kolde R , Laur S, Adler P et al Robust rank aggregation for gene list integration and meta-analysis. Bioinformatics 2012;28:573–80. 22247279 10.1093/bioinformatics/btr709 PMC3278763

[btae536-B8] Lamb J , Crawford ED, Peck D et al The Connectivity Map: using gene-expression signatures to connect small molecules, genes, and disease. Science 2006;313:1929–35. 17008526 10.1126/science.1132939

[btae536-B9] Lin K , Li L, Dai Y et al A comprehensive evaluation of connectivity methods for L1000 data. Brief Bioinform 2020;21:2194–205. 31774912 10.1093/bib/bbz129

[btae536-B10] Liu M , Zhao Z, Wang C et al Harnessing genetic interactions for prediction of immune checkpoint inhibitors response signature in cancer cells. Cancer Lett 2024;594:216991. 38797232 10.1016/j.canlet.2024.216991

[btae536-B11] Lv C , Zeng H-W, Wang J-X et al The antitumor natural product tanshinone IIA inhibits protein kinase C and acts synergistically with 17-AAG. Cell Death Dis 2018;9:165. 29416003 10.1038/s41419-017-0247-5 PMC5833361

[btae536-B12] Samart K , Tuyishime P, Krishnan A et al Reconciling multiple connectivity scores for drug repurposing. Brief Bioinform 2021;22:bbab161. 34013329 10.1093/bib/bbab161 PMC8597919

[btae536-B13] Subramanian A , Narayan R, Corsello SM et al A next generation connectivity map: L1000 platform and the first 1,000,000 profiles. Cell 2017;171:1437–52.e17. 29195078 10.1016/j.cell.2017.10.049 PMC5990023

[btae536-B14] Subramanian A , Tamayo P, Mootha VK et al Gene set enrichment analysis: a knowledge-based approach for interpreting genome-wide expression profiles. Proc Natl Acad Sci USA 2005;102:15545–50. 16199517 10.1073/pnas.0506580102 PMC1239896

[btae536-B15] Tian S , Li Y, Xu J et al COIMMR: a computational framework to reveal the contribution of herbal ingredients against human cancer via immune microenvironment and metabolic reprogramming. Brief Bioinform2023a;24:bbad346. 37816138 10.1093/bib/bbad346 PMC10564268

[btae536-B16] Tian S , Zhang J, Yuan S et al Exploring pharmacological active ingredients of traditional Chinese medicine by pharmacotranscriptomic map in ITCM. Brief Bioinform2023b;24:bbad027. 36719094 10.1093/bib/bbad027

[btae536-B17] Väremo L , Nielsen J, Nookaew I et al Enriching the gene set analysis of genome-wide data by incorporating directionality of gene expression and combining statistical hypotheses and methods. Nucleic Acids Res 2013;41:4378–91. 23444143 10.1093/nar/gkt111 PMC3632109

[btae536-B18] Yang C , Zhang H, Chen M et al A survey of optimal strategy for signature-based drug repositioning and an application to liver cancer. Elife 2022;11:e71880. 35191375 10.7554/eLife.71880 PMC8893721

[btae536-B19] Yang W , Soares J, Greninger P et al Genomics of drug sensitivity in cancer (GDSC): a resource for therapeutic biomarker discovery in cancer cells. Nucleic Acids Res 2013;41:D955–61. 23180760 10.1093/nar/gks1111 PMC3531057

[btae536-B20] Zhang S-D , Gant TW. A simple and robust method for connecting small-molecule drugs using gene-expression signatures. BMC Bioinformatics 2008;9:258. 18518950 10.1186/1471-2105-9-258 PMC2464610

[btae536-B21] Zhang Z , Zhou L, Xie N et al Overcoming cancer therapeutic bottleneck by drug repurposing. Sig Transduct Target Ther 2020;5:25. 10.1038/s41392-020-00213-8 PMC7331117 32616710

