# Supplementary material for: Signature Search Polestar: a comprehensive drug repurposing method evaluation assistant for customized oncogenic signature
Source: Bioinformatics. 2024 Aug 30;40(9):btae536. doi: 10.1093/bioinformatics/btae536 (PMC11398873; doi:10.1093/bioinformatics/btae536)
Supplement: btae536_Supplementary_Data [file btae536_supplementary_data.zip › Supplementary Document V20240820.docx]

**Supplementary Document**

**A comprehensive user manual and a case study using SSP**

**Table of Content**

[**1. How to start?** 1](#_Toc169456171)

[**2. Prepare input data** 3](#_Toc169456172)

[**3. Operation in Benchmark Module** 5](#_Toc169456173)

[**4. Operation in Robustness Module** 7](#_Toc169456174)

[**5. Operation in Application Module** 8](#_Toc169456175)

[**6. Operation in other modules** 12](#_Toc169456176)

[**7. Explanation of methods used in module** 14](#_Toc169456177)

[**8. A case study of liver cancer using SSP** 18](#_Toc169456178)

[**9. Reference** 22](#_Toc169456179)

[**10. Glossary** 24](#_Toc169456180)

**1. How to start?**

A live version of SSP is hosted at **https://web.biotcm.net/SSP/**.


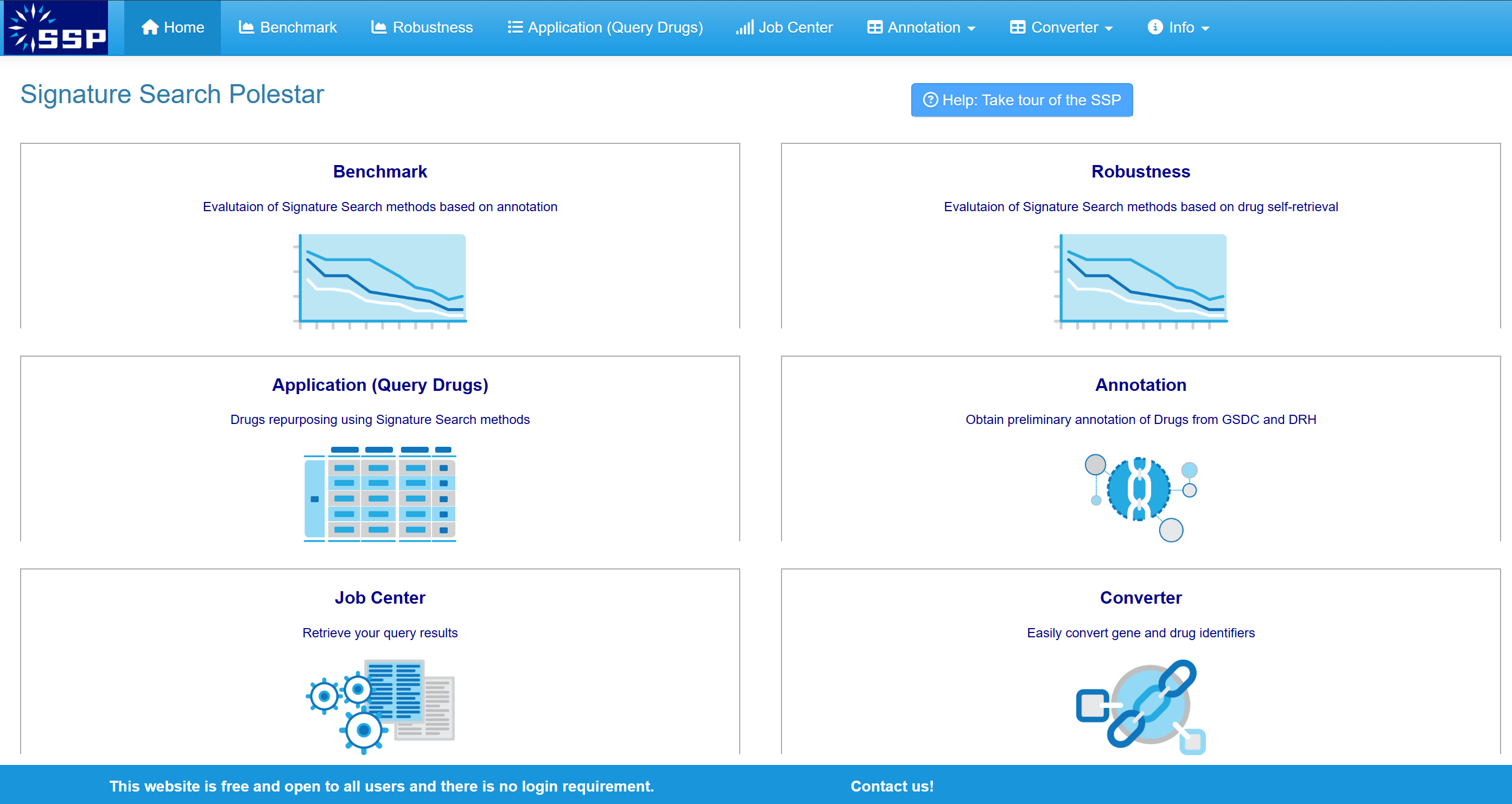


**Figure S1** Homepage of SSP

If you want to deploy SSP on your server, please visit the SSP website (**https://web.biotcm.net/SSP/**), then visit the info-help page to get a full installation of SSP (~3G) (**Figure S2A**). In addition, we also provide source code on https://gitee.com/auptz/benchmark-ss (Chinese) or https://github.com/AuPtZ/BenchmarkSS (English) and download all files and run the “app.R” in RStudio (**Figure S2B**). Notably, essential packages need to be installed before you run.


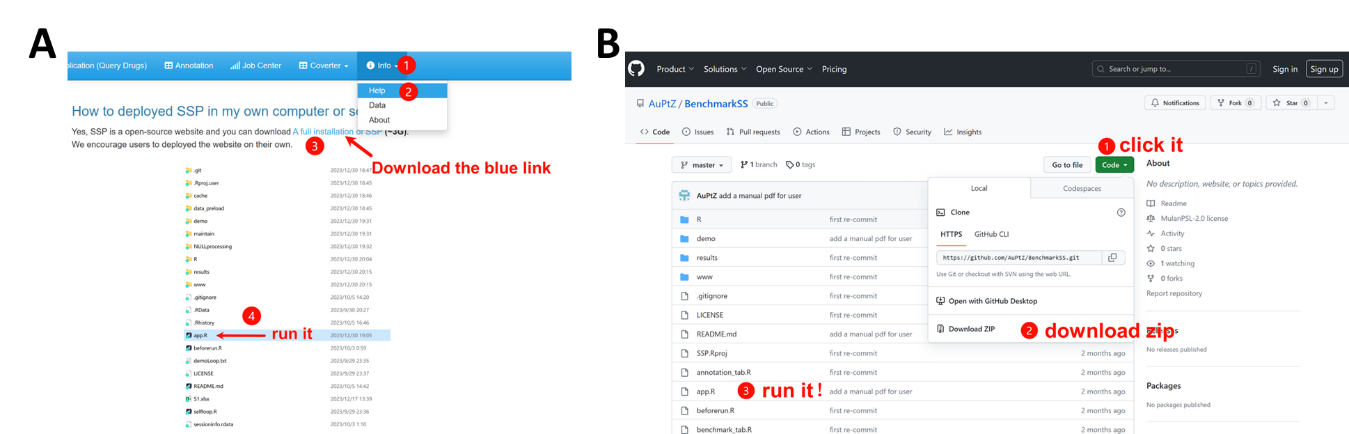


**Figure S2** Two ways to deploy SSP. (A) a full installation on the SSP website. (B) a mirror repository (only source code) on GitHub.

**Important notice for new visit**: As SSP is hosted on the Shiny Server, there is a latency period while the server initializes a session for new users. During this initialization phase, server-side packages are gradually loaded and most reactive widgets may become unresponsive, a common occurrence that can impact the user experience. To mitigate this issue, a pop-up window has been implemented to indicate when the server initialization is complete. It is recommended that users wait until the window closes. Certainly, if a user is currently accessing the SSP, and a new user attempts to access it, the SSP will directly invoke the previous initialization without displaying a pop-up window.


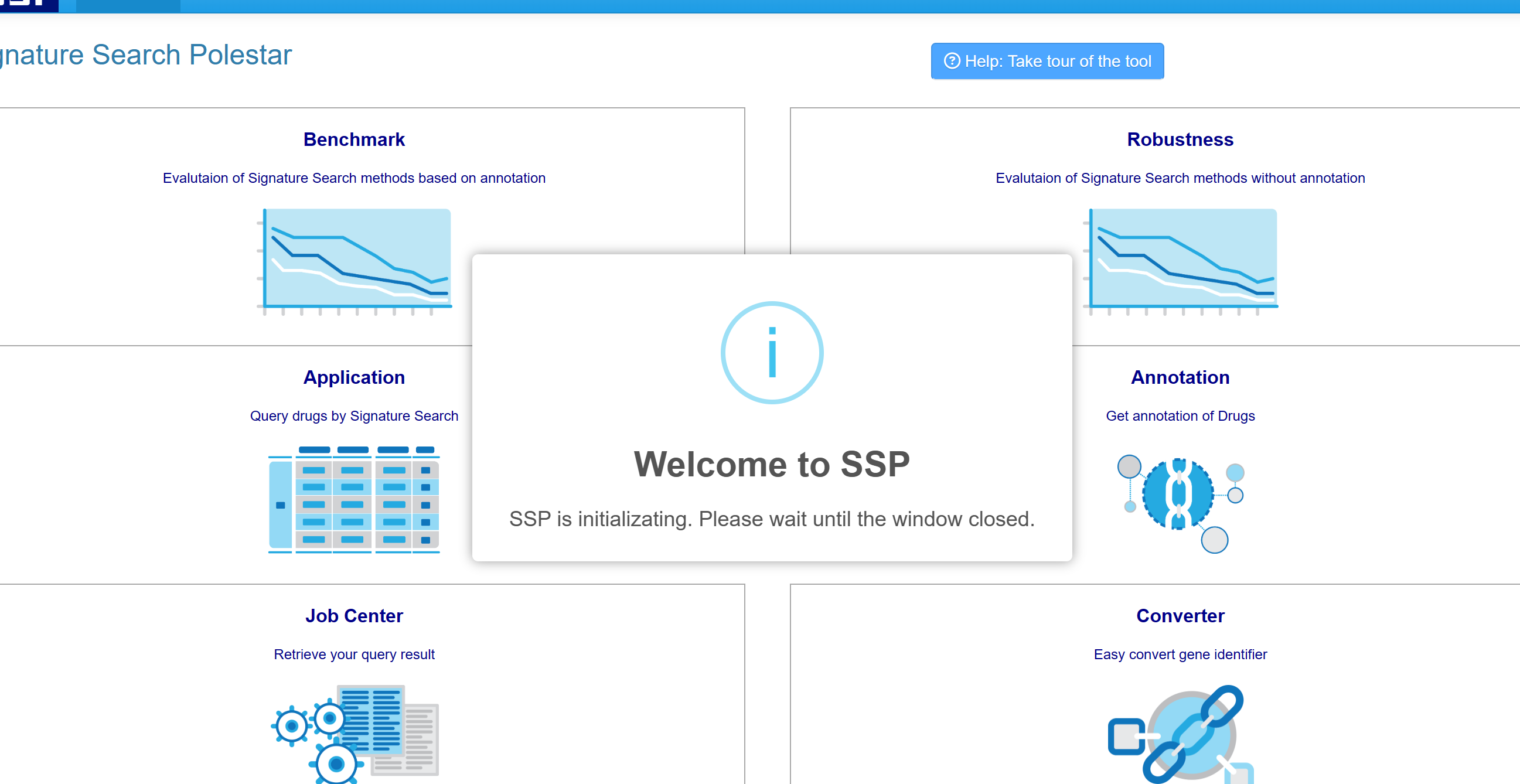


**Figure 3** Pop-up window of initialization when new user visits.

**2. Prepare input data**

SSP requires Two types of data and a selection of dataset:

- An oncogenic signature, header with Gene and log2FC (**Figure S4A**). It typically consists of differentially expressed genes (DEGs) derived from sequencing samples of cell or animal experiments, or patient cohorts, such as GEO, TCGA, and ICGC.

The oncogenic signature should contain at least 10 genes exhibiting a log2FC > 0 and 10 genes exhibiting a log2FC < 0. **Notably, the genes provided by the user should be in the format of the gene symbol and are statistically significant (adjust p-value < 0.05 or FDR < 0.05), ensuring the significance of further analysis.** Should your oncogenic signature contain genes formatted with alternative identifiers (such as EntrezID, Ensembl, UniProt, Gene name, etc.), proceed to the Converter module (for Gene) for the necessary conversion. **Please note that the Converter module only returns genes that are within the pharmacotranscriptomic datasets. In the current version, we have adopted the established practice of utilizing the 978 landmark genes from the LINCS L1000 database, as seen in prior research** (Chen *et al.*, 2017; Yang *et al.*, 2022)**.**

- Drug annotations for AUC and ES (**Figure S4B** and **Figure S4C**) and users must use at least one method to assess the performance of SSM in Benchmark.

The Drug annotations for AUC should comprise a minimum of 50 drugs, and for ES should comprise a minimum of 10 drugs.

Drug annotations are commonly sourced from databases and resources such as ChEMBL, PubChem, scientific literature, clinical trials, and DrugBank. Users have two options: ① Download a blank annotation table and label it manually (**Figure S4D**), or ② Independently compile annotations from various sources and upload them into the Converter module to get a format-compatible annotation file (**Figure S4E**). Converter module (for Drug) could convert drugs with other identifiers or try to correct the drug name (capitalization or the presence of spaces, hyphen, as illustrated in demo1 button) into the acceptable format (drug name in LINCS L1000). In addition, the Converter module accepts the input in the one-column tab-separated file (for ES) or two-column tab-separated file (for AUC) and will keep the second column in the output. **Please note that the Converter module only returns drugs that are within the pharmacotranscriptomic datasets.**

It is impractical to annotate all drugs; however, the more annotations obtained, the more accurate the results will be.


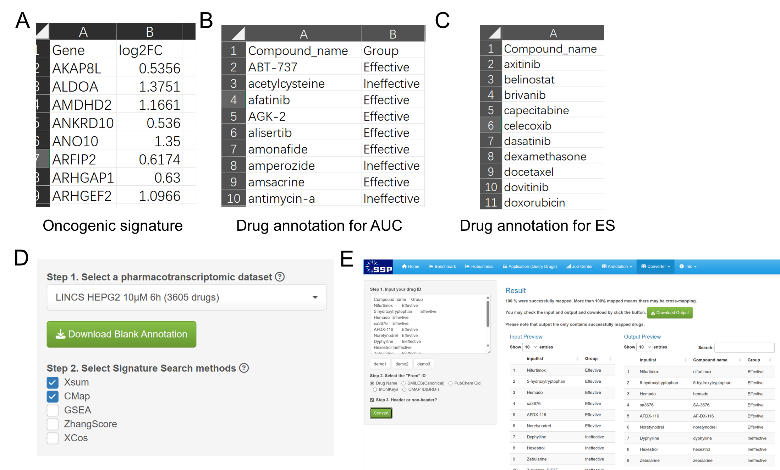


**Figure S4** Demo input in SSP

- In addition, SSP also requires the user to select a built-in pharmacotranscriptomic dataset to perform a job. pharmacotranscriptomic datasets are originally sourced from LINCS L1000 (GSE92742) and are presented in an n × m matrix where n represents the drug names and m denotes a list of gene symbols along with their corresponding log2FC values.


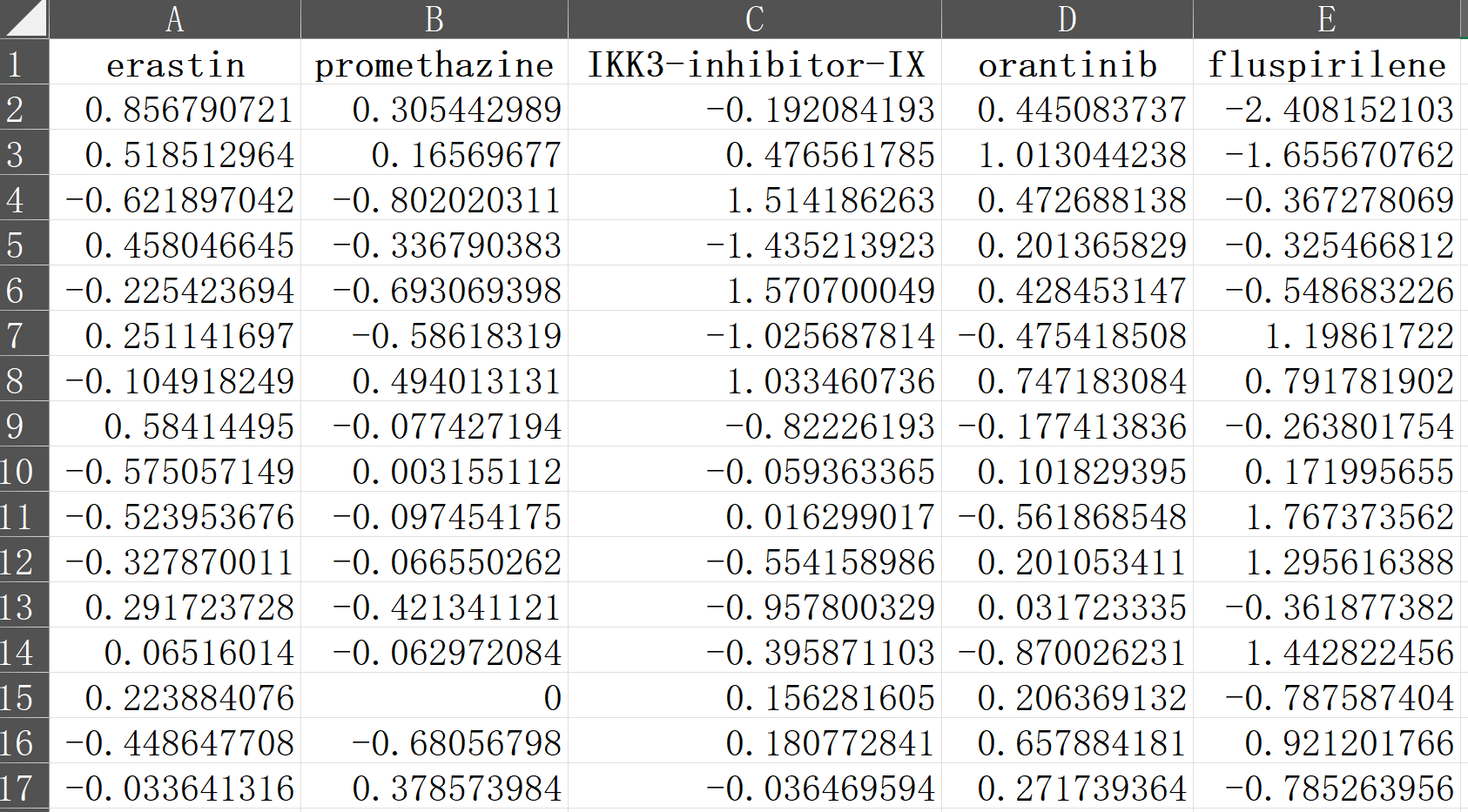


**Figure S5** The matrix of the pharmacotranscriptomic dataset.

**3. Operation in Benchmark Module**

In this module, you can evaluate signature search methods (SSMs) based on signature and well-annotated drugs in LINCS L1000.


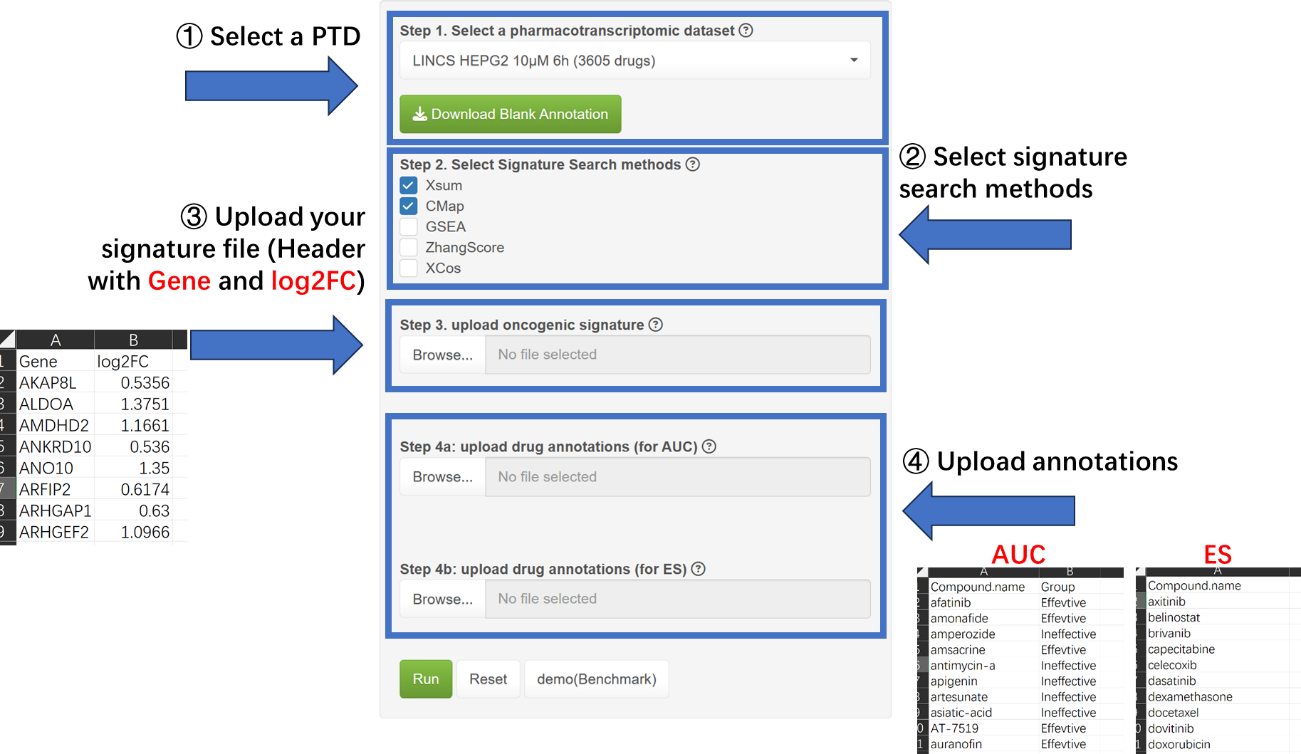


**Figure S6** Workflow in Benchmark module

The Benchmark module, as shown in **Figure S6**, requires the following steps:

- Select pharmacotranscriptomic dataset (LINCS L1000)
- Select SSMs to test (at least two)
- A signature (header with gene and log2FC) to perform a test
- Drug annotations which user can download a blank annotation table of drugs by clicking the download button

If you have annotations for effective or ineffective LINCS L1000 drugs (generally based on whether IC_50_ < 10μM), you can upload them into step 4a. We will then calculate the drug scores and rank them based on the confusion matrix using the Area Under Curve (AUC), the higher AUC indicates better performance.

If you have annotations for effective LINCS L1000 drugs (generally based on Clinical info, such as FDA-approved drugs), you can upload them into step 4b. We will then calculate the drug scores and perform a GSEA-like enrichment score (ES), the lower ES indicates better performance. (Yang *et al.*, 2022)

Finally, click the Run button, and you will obtain a job ID jobid starting with "BEN" (**Figure S7**). It may take approximately 15 minutes to obtain the results, but you can close the page and input the job ID in the job center for later result inquiries.


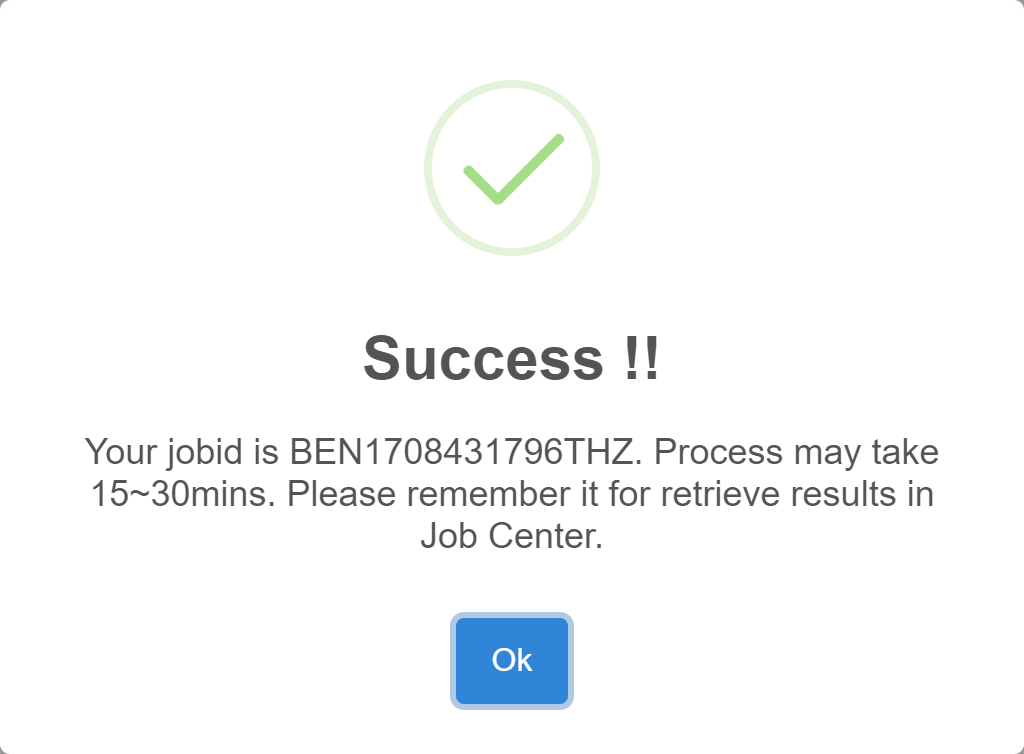


**Figure S7** Pop-up window after successful submission in SSP Benchmark module

The results are displayed in a scatter plot, as shown in **Figure S8**, you can hover over each point to view the specific evaluation score and the corresponding TopN parameter. Each plot is followed with a table and the best SSM and TopN are presented in yellow. As seen in the demo results, the XSum method has a higher score in AUC and a lower score in ES. Scores for AUC perform well at around the TopN 80 and scores for ES perform well at around the TopN 25.


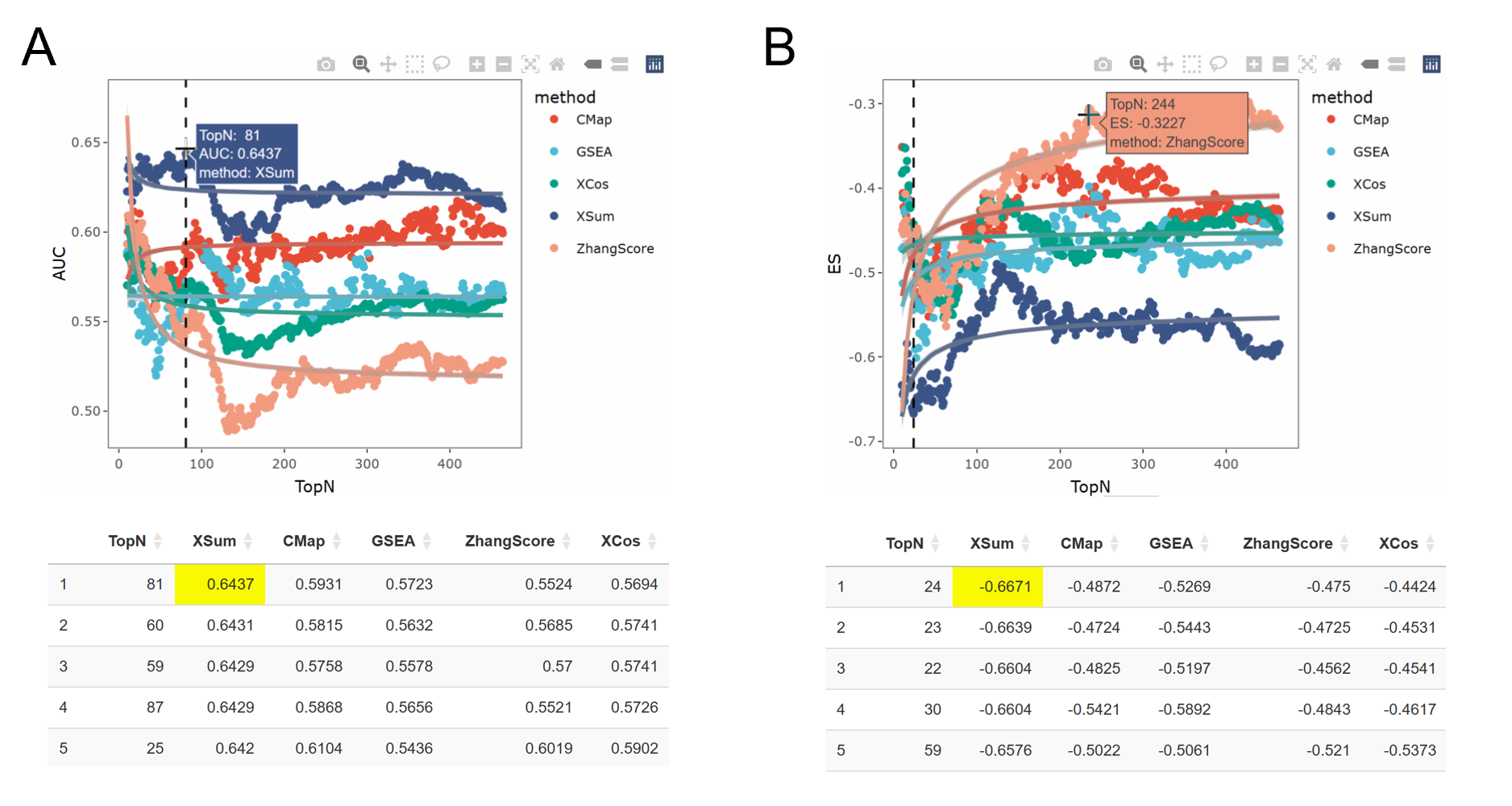
**Figure S8** The result of AUC and ES in the Benchmark module

**4. Operation in Robustness Module**

In this module, you can evaluate the performance of signature search methods (SSMs). In the Benchmark module, we tested SSMs based on drug annotations. However, it may not be appropriate when there is insufficient drug annotation in the pharmacotranscriptomic dataset, such as subtype cancer or rare cancer. Considering that the size of signatures plays a crucial role in the performance of all SSMs, we have proposed a rigorous and robust analysis approach to determine the optimal number of genes in oncogenic signatures (Tian *et al.*, 2023).

The Robustness module, as shown in **Figure S8**, requires the following steps:

- Select a pharmacotranscriptomic dataset (LINCS L1000)
- Select SSMs
- Click “run” button and the results are presented in the right panel


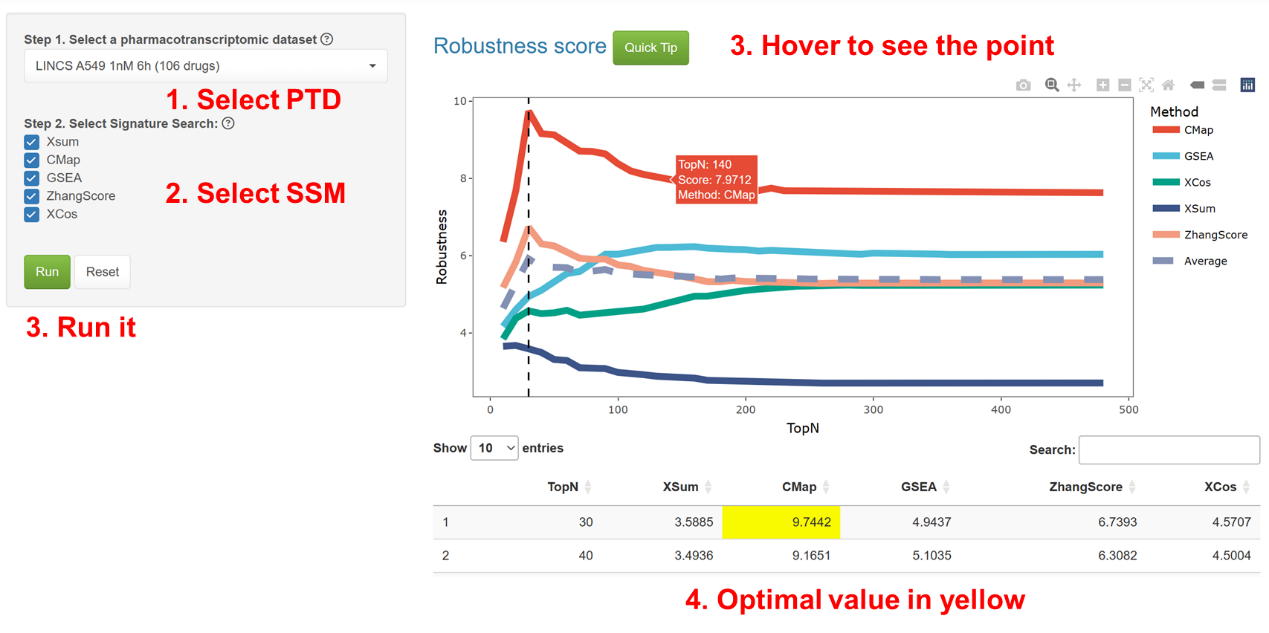


**Figure S9** Demo operation in Robustness module

As seen in the demo results, the CMap method has a higher Rscore at around TopN 30, and CMap, GSEA, and ZhangScore are above the average line, which means they are better and can be combined in the Application module.

Please note that optimal SSM and TopN in Robustness module may differ from that in the Benchmark module. Researchers are strongly encouraged to utilize the Benchmark module, in alignment with their specific fields of study. Should the number of TopN genes from the Robustness module exceed the length of the oncogenic signature, it is recommended to assess whether the scores obtained from the Robustness module at the corresponding length are close to the optimal values. If not, consideration should be given to replacing the oncogenic signature.

**5. Operation in Application Module**

In this module, you can apply signature search methods (SSMs) and TopN to query drugs based on the oncogenic signature input.

An oncogenic signature, header with Gene, and log2FC. It typically consists of differentially expressed genes (DEGs) derived from sequencing samples of cell or animal experiments, or patient cohorts, such as GEO, TCGA, and ICGC.

The oncogenic signature should contain at least 10 genes exhibiting a log2FC > 0 and 10 genes exhibiting a log2FC < 0. **Notably, the genes provided by user should be in the format of gene symbol and are statistically significant (adjust p-value < 0.05 or FDR < 0.05), ensuring the significance of further analysis.** Should your oncogenic signature contain genes formatted with alternative identifiers (such as EntrezID, Ensembl, UniProt, Gene name, etc.), proceed to the Converter module (for Gene) for the necessary conversion.

Optimal SSM and TopN are determined in Benchmark and Application, of note, if you use two oncogenic signatures in SS_cross, please make sure both oncogenic signatures share the near or same TopN and optimal SSM.

Application module provides three approaches to drug repurposing (**Figure S9**).

- **Single method**: Query drugs by one of the SSMs, as the traditional drug repurposing way. Typically, abs(logFC) > ±1 is used for filter differential expression genes.
- **SS_cross**: Query drugs by two oncogenic signatures, and rank them by overall scores. SS_cross aims to find drugs sharing consensus between multiple signatures.
- **SS_all**: Query drugs in multiple SSMs (with higher performance in Benchmark and Application module) and rank them in the same direction (up or down) by robust rank aggregation (RRA), SS_all takes all selected SSM into account and finds the "optimal drugs".


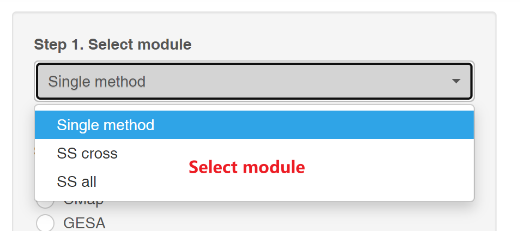


**Figure S10** Method selection in the Application module

**Different way requires different steps:**

For the **Single method**, we need four steps:

① Select a desired SSM,

② Select one pharmacotranscriptomic dataset,

③ Upload your oncogenic signature file (Header with Gene and log2FC), and

④ Set how many TopN genes (up and down) are used, it may be hinted from the Benchmark module or Robustness module.


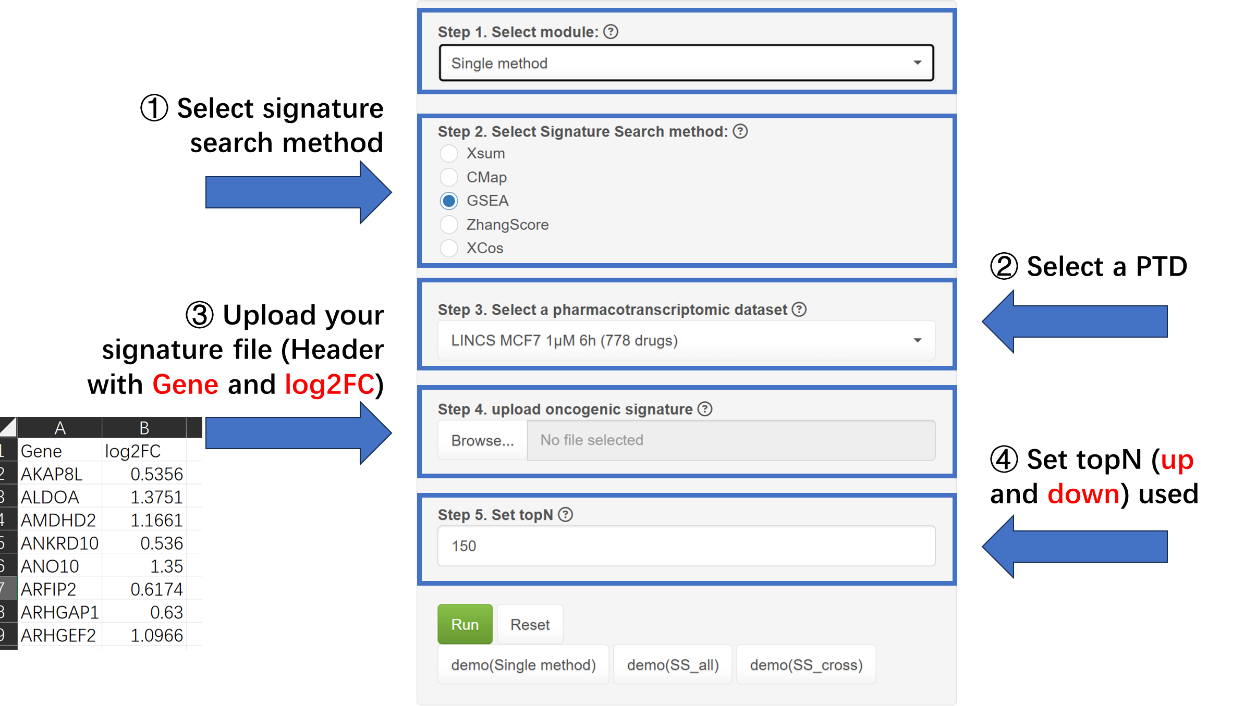


**Figure S11** Usage of a single method in the Application module

For **SS cross**, steps ③ is different:

two oncogenic signature files and their names are required, The name of the first signature represents the X-axis and the second Y-axis in the result figure.


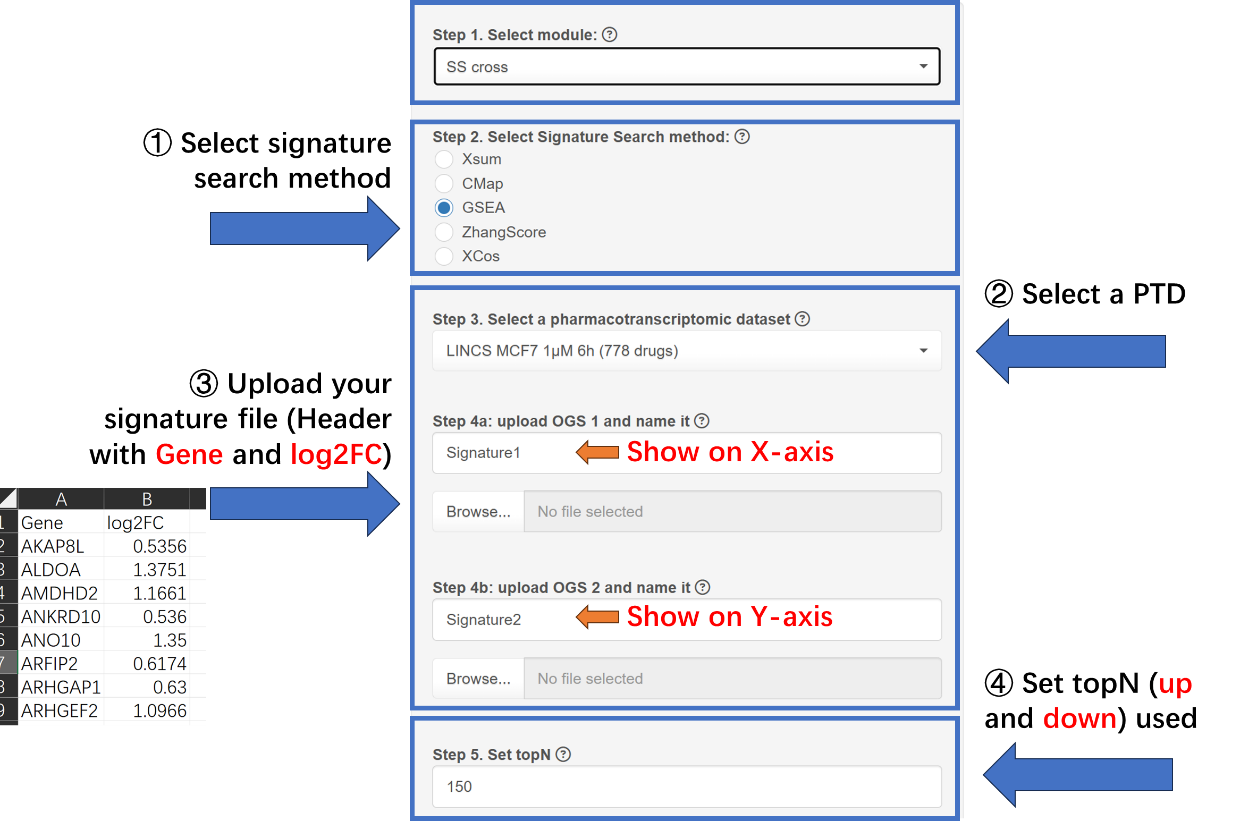


**Figure S12** Usage of SS_cross in Application module

For **SS all**, steps ① is different:

We can select some methods and one direction to rank the drugs, generally, as SSP uses oncogenic signature, please choose “**down**”.


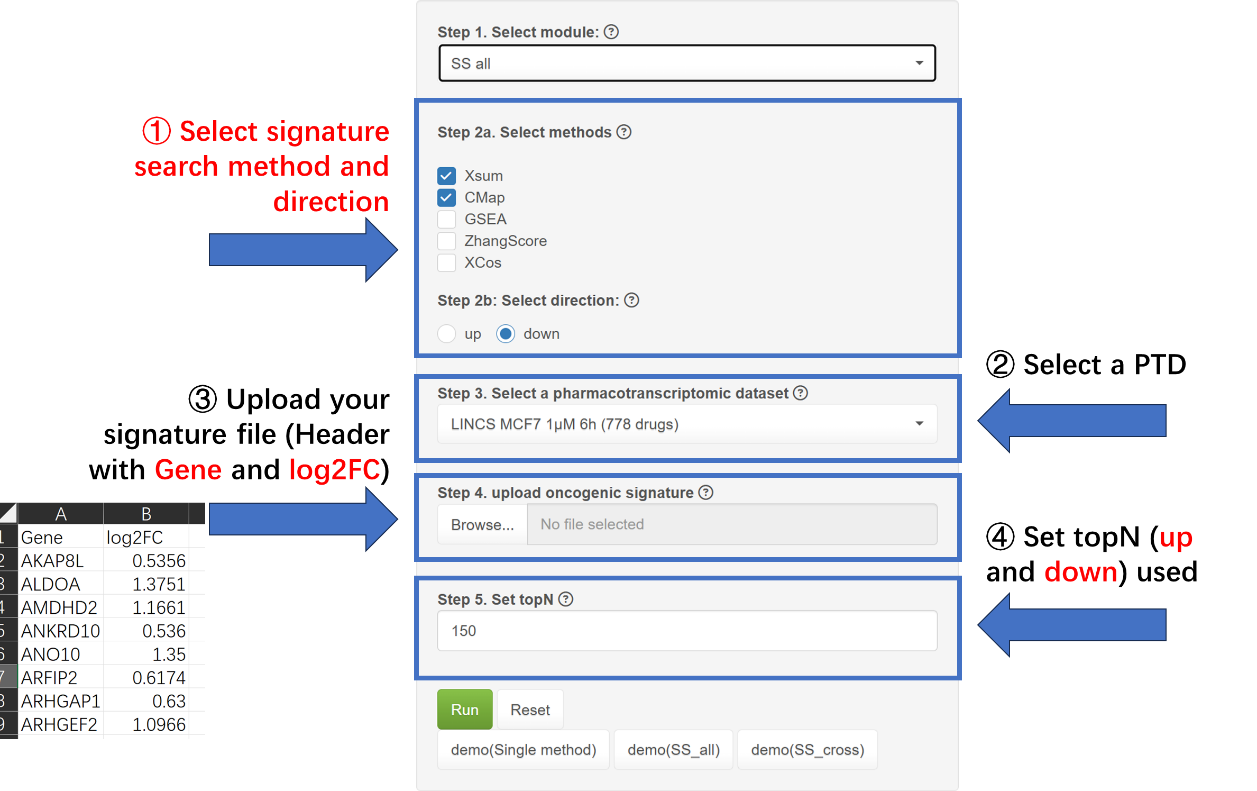


**Figure S13** Usage of SS_all in the Application module

Finally, click the Run button and you will get a **jobid**. It may take 15 minutes to get results, but don't worry, you can close the page and input **jobid** in the job center for result inquiry later.


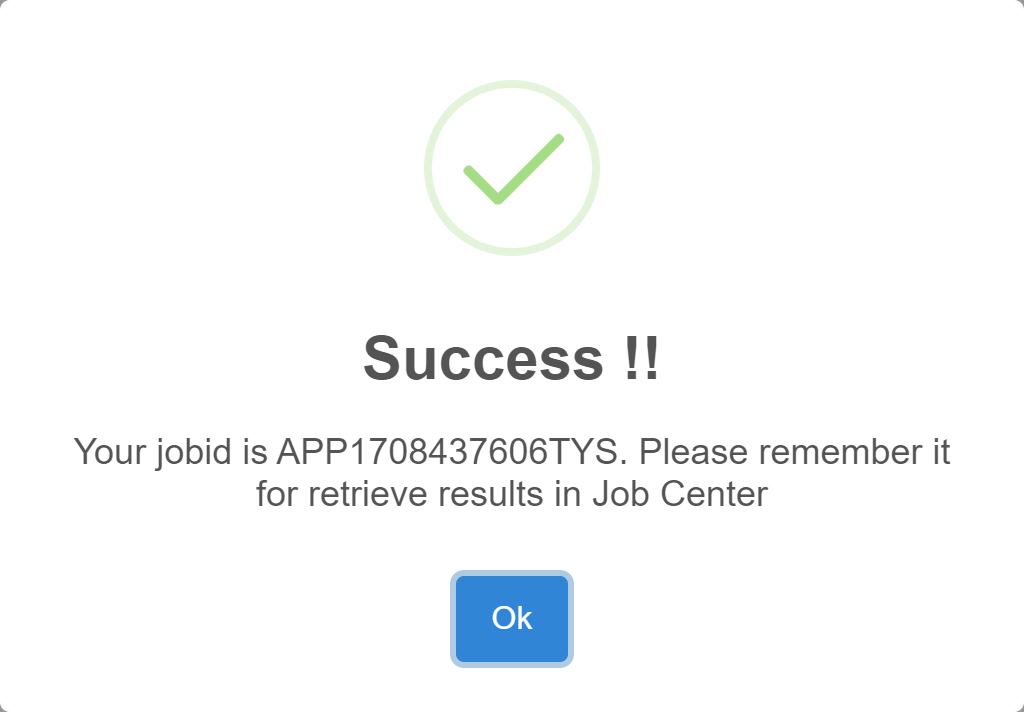


**Figure S14** Successful submission window in the SSP Application module

The results of the Application module are presented in **Figure S14**.


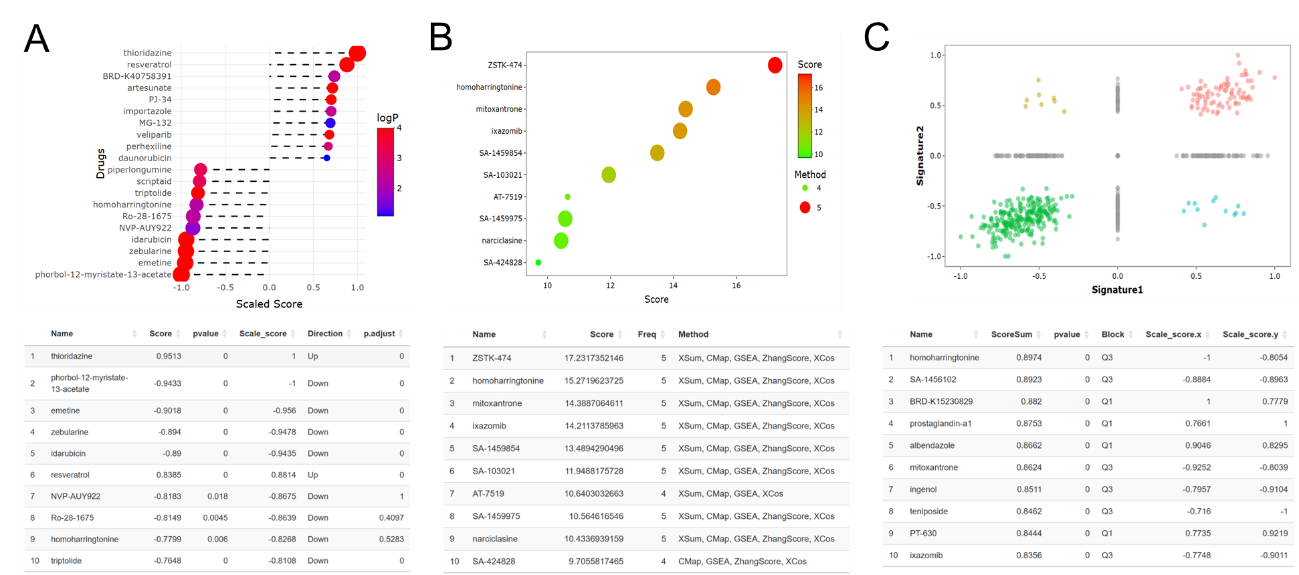


**Figure S15** Results in the Application module

**Figure S15A** illustrates the results of the single method using a lollipop chart, showing the top 5 drugs with the highest positive scores and the top 5 drugs with the highest negative scores. Positive values indicate drugs with a potentially activating effect on the disease, while negative values indicate drugs with a potentially therapeutic effect. The color represents the p-value, and the size of the bubbles is proportional to the absolute value of the scores.

**Figure S15B** demonstrates the results of SS_all, displaying the top 10 drugs with significant scores computed by RRA in the same direction (Up or Down). The top drug is more likely to be promising as it is also the highest-ranked drug in most SSMs. The color represents the number of methods enriched, and the size of the bubbles is correlated with the negative logarithm of the scores.

**Figure S15C** showcases the results of SS_cross, where all drugs are plotted in a scatter plot based on their scores on the x-y plane. The scores are normalized from -1 to 1. Different quarters represent different effects of the drug on the disease. For example, quarter 3 (the lower left corner) indicates that the drug is therapeutic for both diseases.

In addition, a “Quick Tip” button is provided above the plot and the user can click to see a detailed explanation of the plot and column of the table.

**6. Operation in other modules**

***6.1 Annotation***

SSP provides the preliminary drug annotation to facilitate the user’s manual drug annotation.

For AUC, this module integrates annotation data for 286 drugs in 30 cancers sourced from the GDSC database (Yang *et al.*, 2013). A threshold of 10μM is applied, classifying drugs below this value as effective and those above as ineffective. To address IC_50_ redundancy, the median IC_50_ value for duplicate drugs is utilized to represent their activity. Every cancer type is covered with a minimum of 271 drugs (Supplementary Table S1).

For ES, the module includes indication-based annotation data for 163 FDA-approved drugs across 15 cancer types, curated manually from the DRH database, with a minimum of five drugs annotated per cancer type (Supplementary Table S2). Users can directly download annotation files for the Benchmark module corresponding to ES and AUC.


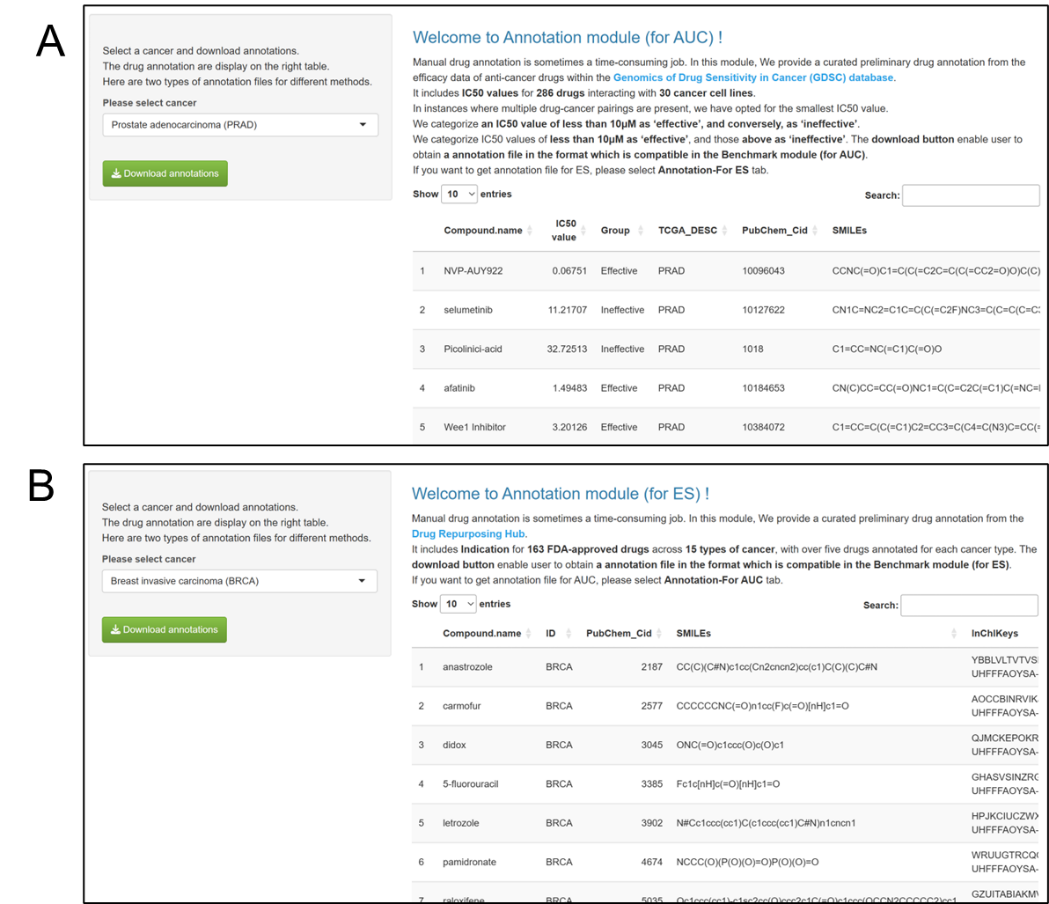


**Figure S16** Webpage of Annotation module.

***6.2 Job center***


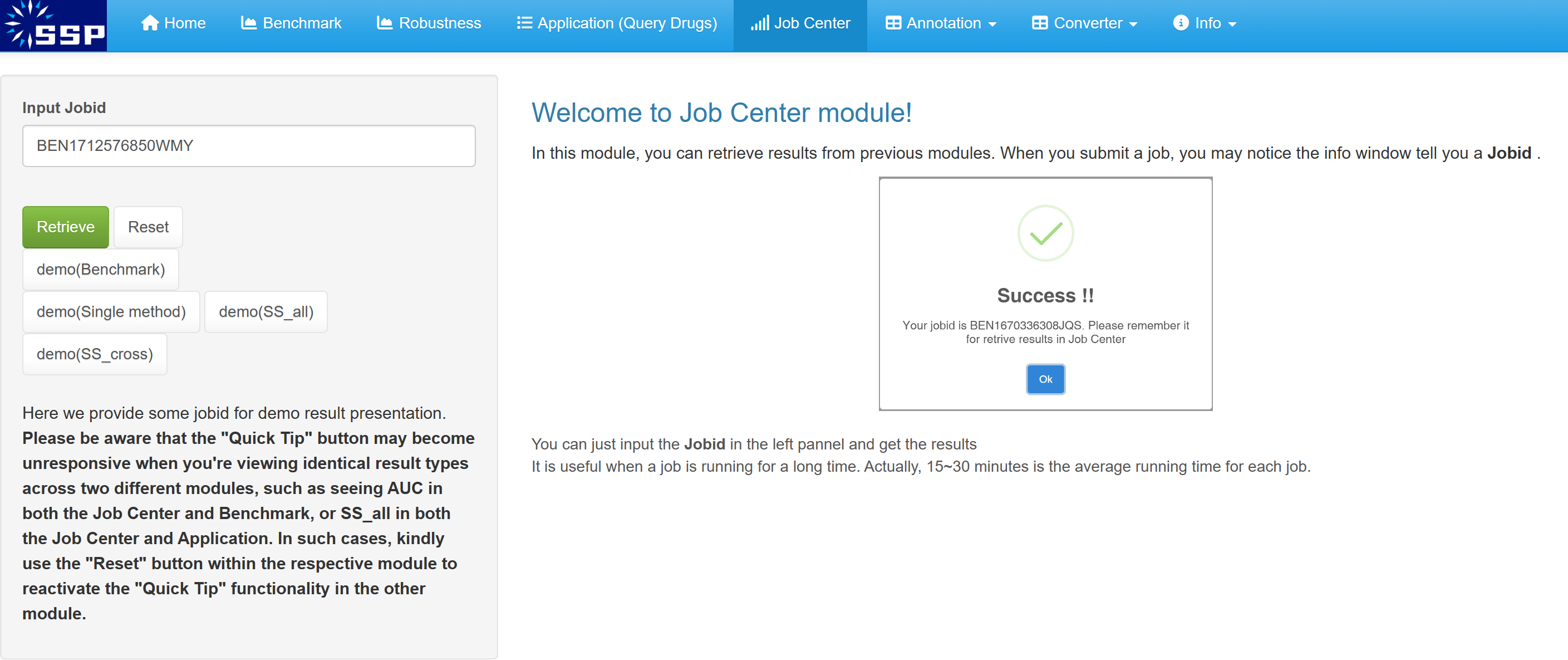


**Figure S17** Webpage of Job center

Considering the large computational workload of the SSP website, we provide a jobid for each task execution, which allows users to retrieve the computational results of their submitted jobs. Users can enter the job id in the job center to view the results of their previous job submissions.

***6.3 Data page***


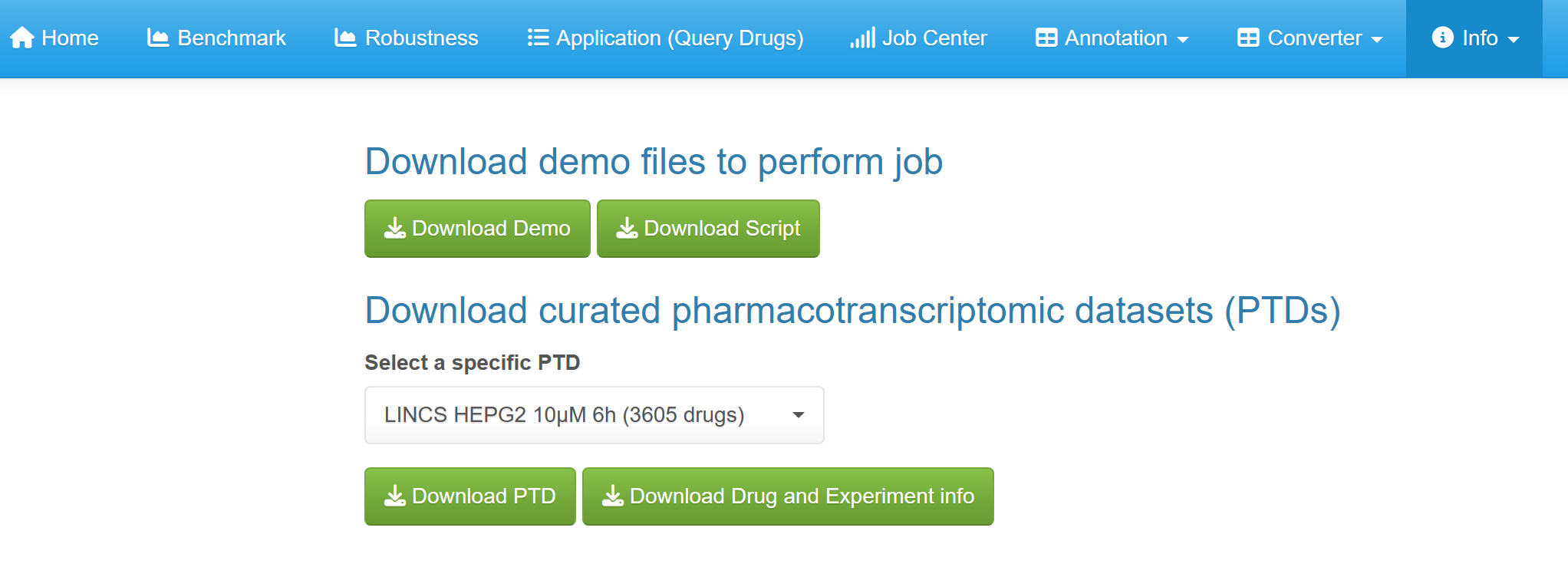


**Figure S18** Webpage of Data

The data page provides the demo file, scripts, and curated pharmacotranscriptomic dataset based on concentration and cell line. Users can download by clicking the corresponding button.

**7.** **Explanation of methods used in modules**


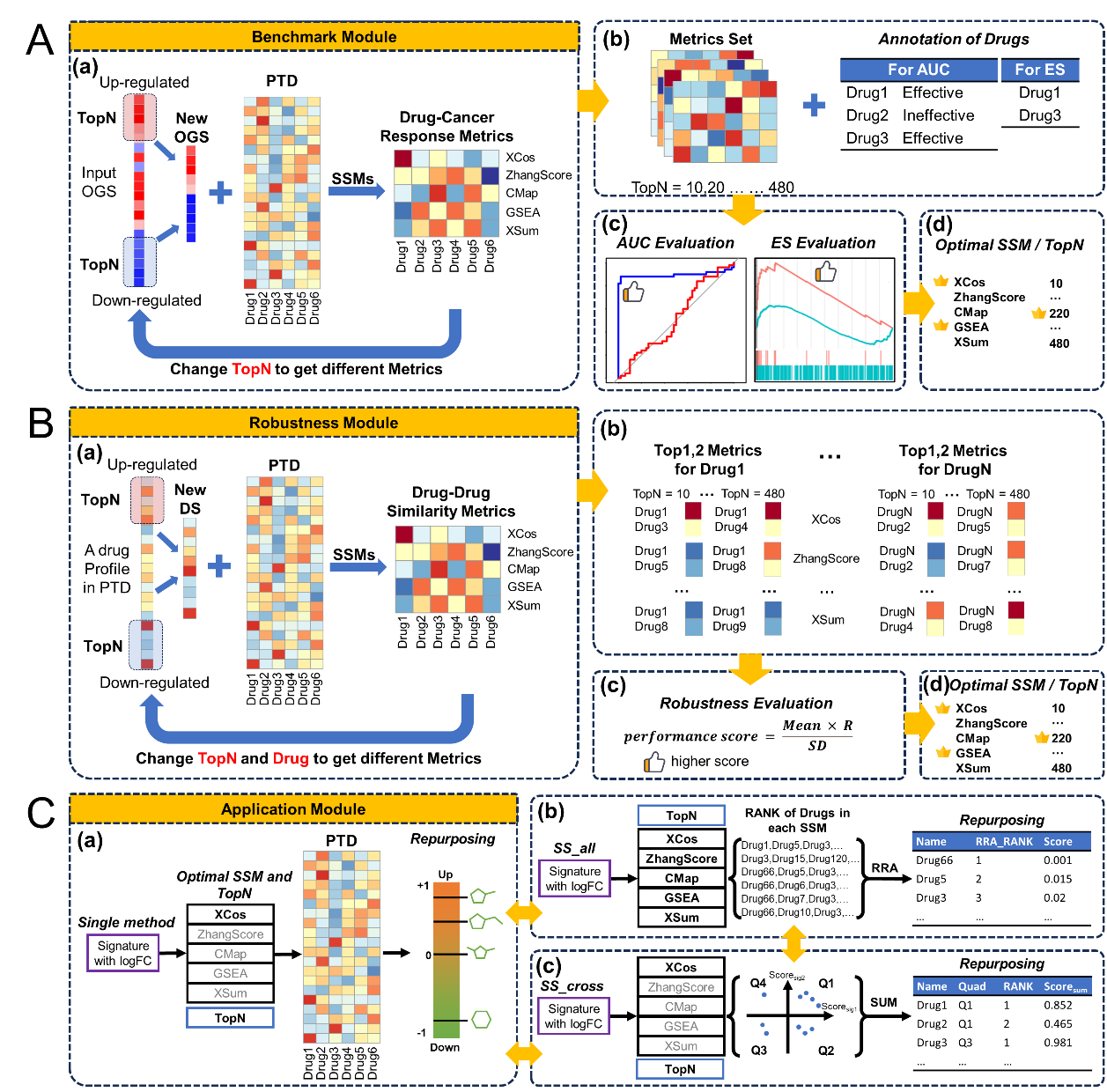


**Figure S19** An illustration of methodologies in SSP. **(A)** Workflow in Benchmark module. (Aa) The input signature is segmented into a new signature (OGS) based on the size of TopN, followed by the calculation of scores between OGS and PTD using SSM, resulting in drug-cancer response metrics. These are represented in a matrix format, where each cell indicates the score of a drug in PTD for OGS under a specific SSM. (Ab) By changing TopN to obtain a set of drug-cancer response metrics, we then proceed to utilize drug annotations, (Ac) Two indexes, AUC and ES, are used to evaluate the performance of each SSM at different TopN levels, and (Ad) identify the most suitable TopN and SSM. **(B)** Workflow in Robustness module. (Ba) We extract and rank the gene expression values of a drug from PTD, segmenting it into a new DS like OGS. We then calculate the scores between DS and all drugs in PTD using SSM, resulting in Drug-DS metrics. These are represented in a matrix format in the figure, where each cell indicates the score of a drug in PTD for DS under different SSMs. (Bb) Subsequently, by varying TopN and SSM, we obtained drug-drug metrics for each drug's signature derived from its expression profile under different SSMs and TopN values within the pharmacotranscriptomic dataset. We then identified the top two scoring drug-drug metrics. (Bc) We assess the performance of each SSM at different TopN levels using a performance score, and (Bd) identify the most suitable TopN and SSM. **(C)** Workflow in Application module. The Application module encompasses three drug repurposing methods: single method, SS_all, and SS_cross. The single method represents the traditional drug repurposing way, which screens drugs based on a single signature with an optimized SSM and TopN. SS_all aggregates multiple optimal SSMs, while SS_cross enables drug screening using two distinct signatures. Further details on the methodologies in SSP can be found in subsequent sections. **Abbreviation**: **DS**, drug signature; **OGS**, oncogenic signature; **PTD**, pharmacotranscriptomic datasets. **TopN**, a top number of up-regulated and down-regulated genes. **SSM**, signature search method.

***7.1 Signature Search Methods***

XSum (Cheng *et al.*, 2014), CMap (Lamb *et al.*, 2006), GSEA (Subramanian *et al.*, 2005), ZhangScore (Zhang and Gant, 2008), and XCos (Cheng *et al.*, 2013) are state-of-the-art SSMs, derived from articles with a high number of citations. Users can read these articles to understand the principles behind the methods. These in-house scripts refer to the signatureSearch (Duan *et al.*, 2020) and RCSM (Lin *et al.*, 2020) packages.

The rationale behind our choice of these SSMs is as follows:

1. These methods encompass both highly cited, well-established approaches (e.g., CMap and GSEA), and those identified as optimal in benchmark analyses (e.g., XCos, XSum, and ZhangScore) (Cheng *et al.*, 2014; Yang *et al.*, 2022; Lin *et al.*, 2020).
2. These methods support identical input and output formats, ensuring compatibility and ease of integration into SSP workflow.
3. Due to the high computational demands in SSP, these SSMs are amenable to refactoring for parallel processing, critical for the effective management of extensive datasets.
4. In our methodological deliberation, we selected CMap 1.0 over CMap 2.0 (Subramanian et al., 2017) due to the identical core algorithms shared between the two, despite CMap 2.0's introduction of tau scores. The computational demands of calculating these percentage-based tau scores are substantial and may hinder SSP efficiency. Moreover, our focus on drug annotation within individual datasets does not necessitate the comparative scoring system that tau scores provide. Additionally, the tau scores' format is incompatible with the standardized scoring system required for seamless integration with the Application module's SS_cross approach. Therefore, CMap 1.0 meets the computational and biological criteria for our Signaling Pathway Prediction (SSP), offering a scientifically meaningful and operationally efficient solution.
5. Taking following factors into account, we have opted for CMap 1.0 over CMap 2.0 (Subramanian et al., 2017) : (i) the core algorithms of both versions are identical, although CMap 2.0 introduces tau scores. These scores, calculated through a comparative analysis across all pharmacotranscriptomic datasets and presented on a percentage scale, require considerable computational resources that could impede the efficiency of SSP; (ii) our research focuses on drug annotation within individual pharmacotranscriptomic datasets, which does not necessitate a comparative scoring system; (iii) the SS_cross approach in the Application module requires a standardized and easily integrated scoring system, which the percentage-based tau scores may not meet.

***7.2 Benchmark***

As shown in **Figure S19A**, the oncogenic signature in the benchmark undergoes iterative tailored filtration for assessing the performance of SSMs. Specifically, the genes in an oncogenic signature are ordered based on logFC, then an equivalent number (TopN) of significantly up-regulated and down-regulated genes are selected to form a new oncogenic signature. This approach guarantees a comprehensive and balanced representation of the varied gene expression alterations associated with the disease state. Then, five state-of-the-art SSMs, XSum, CMap, GSEA, ZhangScore, and XCos are called to calculate scores. We compute the scores for these SSMs across a range of TopN values (10~480) to obtain drug-cancer similarity metrics. In general, a score greater than 0 indicates that the drug is potentially agonistic to the cancer, while a score less than 0 indicates that the drug is potentially therapeutic to the cancer.

Next, based on the drug annotation data, two benchmarking indices, namely area under the curve (AUC) and enrichment score (ES), are generated for evaluating the performance of drug-disease similarity metrics. The methodologies of AUC and ES are derived from commonly used metrics for evaluating drug efficacy (Yang et al., 2022). Although there are some different benchmarking standards (Luo et al., 2022; Fang et al., 2021), they are significantly different in score computation and require more annotation preparation, which may hinder users from easily using them.

Within the Benchmark module, these benchmarking standards can be adeptly employed to utilize the scores based on pharmacotranscriptomic datasets and annotation information, thereby facilitating an accurate assessment of the precision of SSMs. The AUC uses scores of drugs annotated with “Effective” and “Ineffective” and aims to assess whether the SSM can effectively discriminate between these categories, with **a higher score signifying a more effective method and a suitable TopN**. This metric applies to drug annotations derived from large-scale experimental screenings, such as determining drug efficacy based on IC_50_ values. The ES just like GSEA, aims to evaluate whether the drugs can be enriched at the top of the descending ordered list of all drugs based on scores from SSM, with **a negative score indicating a better method and a suitable TopN**. This metric is suitable for drug annotations based on clinical practice, where a minority of drugs are known to be effective, and the efficacy of the majority remains uncertain.

if you only upload a annotation for ES, we recommend user select SSMs and TopN with lowest scores for later query in Application module. If the SSM in result exhibits monotonic decrease, it is recommended to directly set the length of OGS to TopN for later query in Application module.

if you only upload an annotation for AUC, we recommend user select SSMs and TopN with the highest scores for later queries in the Application module. If the SSM in the result exhibits a monotonic increase, it is recommended to directly set the length of the oncogenic signature to TopN for later query in the Application module.

if you upload both annotations for AUC and ES, we recommend user select SSMs and TopN with relatively high scores in both AUC and ES for later query in the Application module. For example, TopN in the top 10 of results ES and AUC is acceptable.

if you want to use SS_cross, it is important to note that we recommend each oncogenic signature be evaluated in the Benchmark module. If the optimal TopN and SSM for two oncogenic signatures are identical or close (with high scores in the same TopN or a high ranking in SSM), this indicates a strong match. If not, it is advisable to replace the oncogenic signatures. if you want to use SS_all, just select the SSMs with high performance over minority in AUC.

***7.3 Robustness***

As, shown in **Figure S19B**, drugs within the same dataset in the LINCS L1000 dataset were assigned labels ranging from 1 to ***n***. For each drug, the top ***x*** up-regulated and top ***x*** down-regulated (TopN) differentially expressed genes were extracted from its gene expression profile to form a signature. These signatures were then used to query one of the five SSMs, yielding drug-drug metrics across all drugs. Subsequently, by varying TopN and SSM, we obtained drug-drug metrics for each drug's signature derived from its expression profile under different SSMs and TopN values within the pharmacotranscriptomic dataset. We then identified the top two scoring drug-drug metrics. To assess the robustness of these methods across various TopN values, we employed three parameters:

(1) The correlation (R) between the input label and the top-ranked output label for all drugs, for example, drug**1** input with drug**1** top-ranked output, for specific TopN and SSM.

(2) The mean difference between the top two scores (Score_top1_ – Score_top2_) across outputs for a specific TopN and SSM.

(3) Standard deviation (SD) of all differences of top1 and top2 (Score_top1_ – Score_top2_) across outputs for a specific TopN and SSM.

Finally, the Performance score can be expressed by the following formula:

$$Performance score = \frac{Mean \times R}{SD}$$

SSM and TopN can be considered to have achieved satisfactory performance if they can accurately identify the input active drug (stronger correlation), effectively differentiate between drugs (higher difference score), and demonstrate good stability (lower SD). Hence, **a higher score means a better SSM with a suitable TopN**. **In this study,** **performance scores were evaluated for TopN values ranging from 10 to 480 in increments of 10.**

**We recommend user select SSMs and TopN with highest scores for later query in Application module. If the scores continue to increase with increasing TopN, it is recommended to directly set the length of oncogenic signature to TopN for later query in Application module.**

***7.4 SS_cross and SS_all***

As, shown in **Figure S19C**, Two methods, The "SS_all" method and "SS_cross" method were designed to find promising drugs with consensus under multiple SSMs or oncogenic signatures (Tian et al., 2023).

In the SS_all, the user is required to select SSMs with higher performance in Benchmark or Robustness. Then, drug-cancer metrics are generated by all optimal SSMs against an oncogenic signature. The ranks of drugs based on metrics in the same direction (< 0 or > 0) are combined using robust rank aggregation (Kolde et al., 2012). The robust rank aggregation returns a p-value of each drug and we assign -log(P-value) as the overall score to each drug. Hence, a drug with a top rank in more SSMs results in a higher overall score and indicates greater potential to be promising.

In SS_cross, the user is required to prepare two distinct oncogenic signatures and then drug-cancer metrics are generated by these oncogenic signatures with one SSM. These drugs are then divided into four quadrants based on the sign of these metrics (Chen et al., 2021), representing potentially agonistic response (> 0) or therapeutic response (< 0). (Q1: both scores >0, Q2: Score_sig1_ <0 but Score _sig2_ >0, Q3: both scores <0, Q4: Score _sig1_ >0 but Score _sig2_ <0). Then, we calculated a unified score by the square root of absolute values:

$$\begin{aligned} \mathrm{Score}_{\mathrm{sum}}= \sqrt{{abs(Score}_{sig1}\times\mathrm{Score}_{\mathrm{sig}2})} \end{aligned}$$

The drugs that exhibit therapeutic response in both oncogenic signatures with higher Score_sum_ show promise for repurposing, particularly those located in the lower left corner (Q3).

**8. A case study of liver cancer using SSP**

Liver cancer, recognized as one of the deadliest malignancies globally, is among the top three causes of cancer death in 46 countries (Rumgay *et al.*, 2022). The majority of liver cancers are Hepatocellular carcinoma (HCC). Despite the advent and approval of numerous drugs, such as Ramucirumab (Zhu *et al.*, 2019), Pembrolizumab (Finn, Ryoo, *et al.*, 2020), Atezolizumab-Bevacizumab (Finn, Qin, *et al.*, 2020), and Nivolumab-Cabozantinib (Yau *et al.*, 2023). These treatments have generally provided only marginal survival advantages. Consequently, there is an urgent and pressing need for more efficacious therapeutic options to combat HCC.

In this case study, we try to find a promising drug for HCC. **Please note all the signatures for the case study are provided on the Info-data page.**

***8.1 obtain an oncogenic signature***

oncogenic signature is a gene list with log2FC, which is usually generated from an experiment or patient cohorts. In this study, we obtain an HCC-related oncogenic signature from a previous publication (Chen *et al.*, 2017).

Drug annotations were then uploaded to the converter page to prioritize genes present in the LINCS L1000 dataset resulting in 54 genes in down and 19 genes in up. (see **CS_OGS.txt**)

In general, we recommend an oncogenic signature with at least 20 genes.

***8.2 obtain drug annotation***

Drug annotation for AUC was obtained from the Annotation module (drugs in *Liver hepatocellular carcinoma (LIHC)*), and ChEMBL (version 33) (Zdrazil *et al.*, 2024) and Liver Cancer Model Repository (LIMORE) data sets (Qiu *et al.*, 2019). As we keep the minimum IC_50_ values to reduce the redundancy in the Annotation module, precedence was given to the IC_50_ data treated on the HepG2 cell line from ChEMBL and LIMORE during the aggregation process.

Drug annotations for ES were obtained from Clinicaltrail.gov by identifying HCC-specific clinical trials that involve drug interventions and assessing anti-tumor activity.

Drug annotations were then uploaded to the converter page to prioritize drugs present in the LINCS L1000 dataset resulting in 185 drugs in AUC and 29 drugs in ES. (see **CS_ES.txt** and **CS_AUC.txt**). Additionally, within the ES annotation file, only 19 drugs were also present in the AUC annotation file, highlighting the independence of the two annotation sources.

***8.3 perform benchmark, find an optimal method, and TopN***

Considering the focus on HCC and the aim to include a broader range of drugs, we selected the pharmacotranscriptomic dataset specific to the HepG2 cell line (treatment with 10μM for 6 hours).

The files **CS_OGS.txt**, **CS_ES.txt**, and **CS_AUC.txt** were uploaded to the corresponding file input on the benchmark module, selecting "LINCS HEPG2 10μM 6h (3605 drugs)". The task was submitted, and the results (**Figure 20**) indicated that at TopN=23, the XSum method achieved optimal performance in both AUC and ES evaluation metrics (maximizing AUC and minimizing ES). Consequently, that the XSum method with TopN set to 23 was selected as the parameter and approach for drug repositioning.

It is noteworthy that instances where the same TopN ranks first in both evaluation metrics are not usual. Typically, TopN values that appear in the top 10 for both ES and AUC can be recommended for use in the Application module.


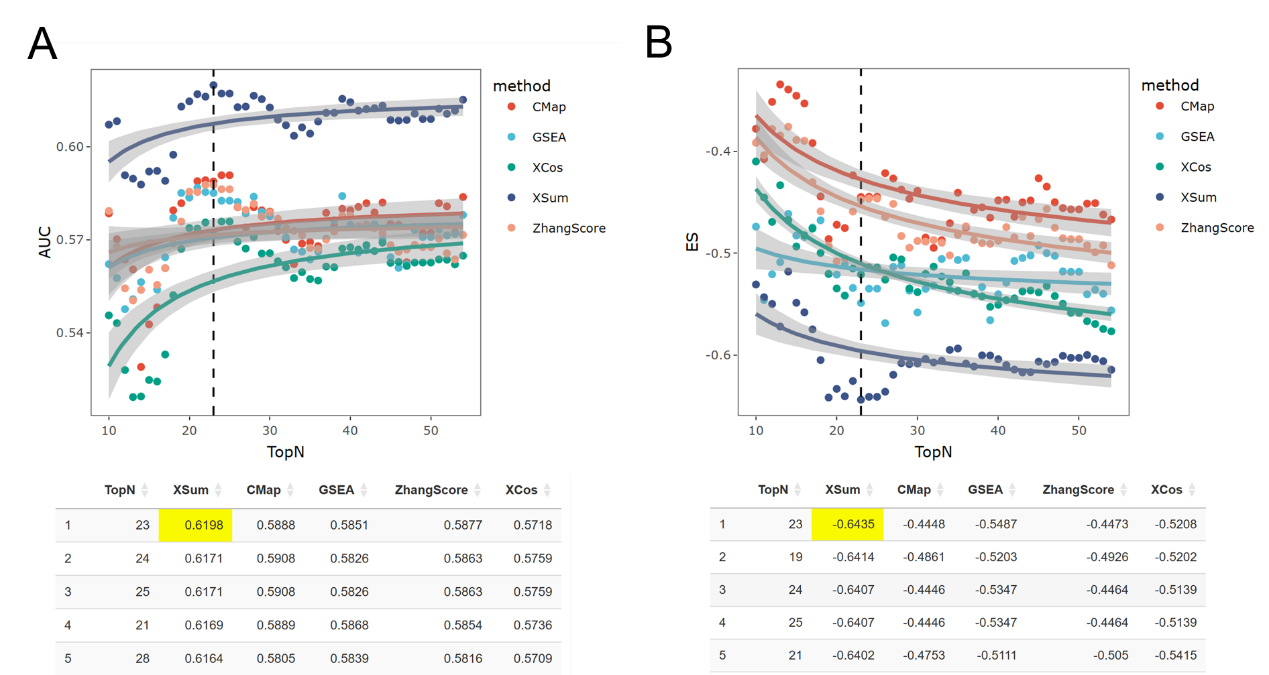


**Figure 20** Result in Benchmark module, XSum and TopN = 23 is the optimal both in AUC and ES. These parameters are used in the Application module.

***8.4 drug repurposing by Single Method in the application module***

In the Application module, the “single method” option was selected, with CS_OGS.txt provided as the oncogenic signature input and TopN set to 23. The result, as depicted in the following figure (**Figure 21**), shows that among the top 10 drugs in the down-regulation (lower left of the figure), LDN-193189, a selective BMP type I receptor inhibitor, has been reported to exhibit anti-HCC effects (Liang *et al.*, 2021), thereby demonstrating the potential of SSP to identify novel uses for existing drugs.


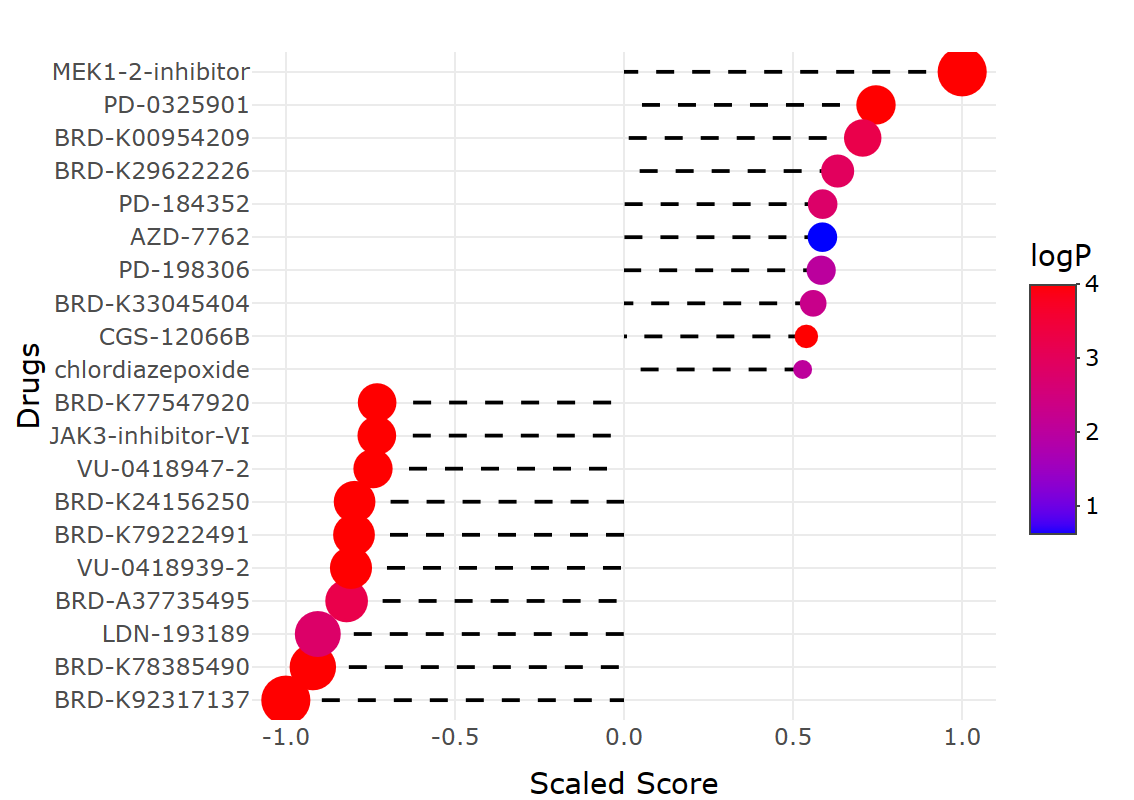


**Figure 21** Result of drug repurposing.

***8.5 further exploration of drug***

To delve deeper into the properties and experimental context of the drug LDN-193189, it is necessary to access comprehensive drug and experimental information beyond what is provided in the figure and table of the Application module. Hence, the SSP offers an Info-data page that allows users to obtain gene expression data and detailed drug information tailored to a specific pharmacotranscriptomic dataset. By selecting “LINCS HEPG2 10μM 6h (3605 drugs)” on the info-data page, users can access and download comprehensive drug and experimental information by clicking the respective link (**Figure 22**).


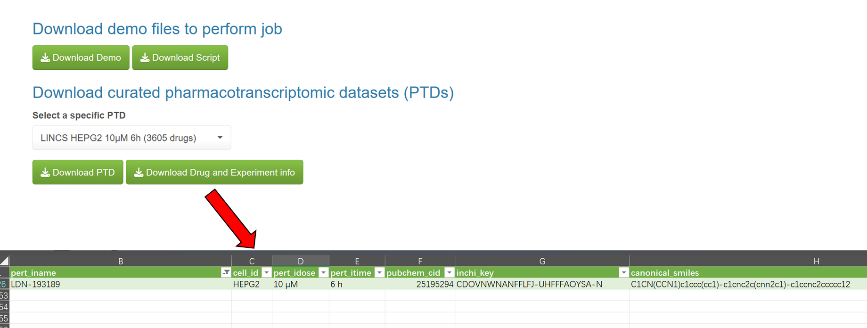


**Figure** 22 Download information about the drug and experiment.

***8.6 a simple illustration for other applicable modules or methods in the case study***

**Robustness module**: The purpose of the Robustness module is to assess the performance of SSMs in scenarios where drug annotations are limited, such as for subtypes of cancer or rare cancers. In this case study, for example, if we want to study Intrahepatic cholangiocarcinoma, a rare subtype of liver cancer, the drug annotations for the "LINCS HEPG2 10μM 6h (3605 drugs)" dataset are quite insufficient. Therefore, users can proceed to the Robustness module to evaluate the performance of each SSM based on drug self-retrieval and to review the pharmacotranscriptomic dataset. The optimal SSM and TopN values are identified in a manner consistent with the Benchmark module. The optimal method and TopN are labeled as in the Benchmark module. It should be noted that the performance of SSMs in the Robustness module may differ from that in the Benchmark module. Researchers are strongly encouraged to utilize the Benchmark module, in alignment with their specific fields of study. Should the number of TopN genes from the Robustness module exceed the length of the oncogenic signature, it is recommended to assess whether the scores obtained from the Robustness module at the corresponding length are close to the optimal values. If not, consideration should be given to replacing the oncogenic signature.

**SS_cross**: When users have two oncogenic signatures derived from different sources, they can upload all of them to the SS_cross by properly naming each oncogenic signature. Typically, the focus is on the third quadrant of the results, where drugs with negative values (<0) on both the X-axis and Y-axis. It is important to note that we recommend each oncogenic signature be evaluated in the Benchmark module. If the optimal TopN and SSM for two oncogenic signatures are identical or close (with high scores in the same TopN or a high ranking in SSM), this indicates a strong match. If not, it is advisable to replace the oncogenic signatures. For the plot result, we recommend that users download and annotate figures themselves to identify their drugs of interest. It is worth noting that SSP employs *plotly* package for interactive figures, allowing users to hover their mouse over points to view details of drugs, thus facilitating exploration.

**SS_all**: When evaluating with the Benchmark module and finding that the performance of SSM is closely aligned, making it difficult to select the most appropriate one, it is advisable to consider all the desirable SSMs that exhibit good performance. In this case study, except for XSum, the performance of the other four SSMs is very close, so if XSum is the worst, we can combine the four other SSMs in SS_all. In addition, if we use the Robustness module to determine SSM, SSM over the average line is highly recommended used in SS_all. Please note that SSP with poor performance in the Benchmark and Robustness module is not recommended for inclusion, as it may adversely affect the outcomes. By uploading the oncogenic signature and selecting multiple SSMs that perform well, the results will integrate the outcomes of all considered SSMs and present them accordingly. Typically, the drugs that rank highest in this analysis are often the most promising.

**9. Reference**

Chen,B. *et al.* (2017) Reversal of cancer gene expression correlates with drug efficacy and reveals therapeutic targets. *Nat Commun*, **8**, 16022.

Chen,S. *et al.* (2021) The phytochemical hyperforin triggers thermogenesis in adipose tissue via a Dlat-AMPK signaling axis to curb obesity. *Cell Metab*, **33**, 565-580.e7.

Cheng,J. *et al.* (2013) Evaluation of analytical methods for connectivity map data. *Pac Symp Biocomput*, 5–16.

Cheng,J. *et al.* (2014) Systematic evaluation of connectivity map for disease indications. *Genome Med*, **6**, 540.

Duan,Y. *et al.* (2020) signatureSearch: environment for gene expression signature searching and functional interpretation. *Nucleic Acids Res*, **48**, e124.

Finn,R.S., Qin,S., *et al.* (2020) Atezolizumab plus Bevacizumab in Unresectable Hepatocellular Carcinoma. *N Engl J Med*, **382**, 1894–1905.

Finn,R.S., Ryoo,B.-Y., *et al.* (2020) Pembrolizumab As Second-Line Therapy in Patients With Advanced Hepatocellular Carcinoma in KEYNOTE-240: A Randomized, Double-Blind, Phase III Trial. *J Clin Oncol*, **38**, 193–202.

Kolde,R. *et al.* (2012) Robust rank aggregation for gene list integration and meta-analysis. *Bioinformatics*, **28**, 573–580.

Lamb,J. *et al.* (2006) The Connectivity Map: using gene-expression signatures to connect small molecules, genes, and disease. *Science*, **313**, 1929–1935.

Liang,Z. *et al.* (2021) The binding of LDN193189 to CD133 C-terminus suppresses the tumorigenesis and immune escape of liver tumor-initiating cells. *Cancer Lett*, **513**, 90–100.

Lin,K. *et al.* (2020) A comprehensive evaluation of connectivity methods for L1000 data. *Briefings in Bioinformatics*, **21**, 2194–2205.

Qiu,Z. *et al.* (2019) A Pharmacogenomic Landscape in Human Liver Cancers. *Cancer Cell*, **36**, 179-193.e11.

Rumgay,H. *et al.* (2022) Global burden of primary liver cancer in 2020 and predictions to 2040. *J Hepatol*, **77**, 1598–1606.

Subramanian,A. *et al.* (2017) A Next Generation Connectivity Map: L1000 Platform and the First 1,000,000 Profiles. *Cell*, **171**, 1437-1452.e17.

Subramanian,A. *et al.* (2005) Gene set enrichment analysis: A knowledge-based approach for interpreting genome-wide expression profiles. *Proceedings of the National Academy of Sciences*, **102**, 15545–15550.

Tian,S. *et al.* (2023) Exploring pharmacological active ingredients of traditional Chinese medicine by pharmacotranscriptomic map in ITCM. *Brief Bioinform*, **24**, bbad027.

Yang,C. *et al.* (2022) A survey of optimal strategy for signature-based drug repositioning and an application to liver cancer. *eLife*, **11**, e71880.

Yang,W. *et al.* (2013) Genomics of Drug Sensitivity in Cancer (GDSC): a resource for therapeutic biomarker discovery in cancer cells. *Nucleic Acids Res*, **41**, D955-961.

Yau,T. *et al.* (2023) Nivolumab Plus Cabozantinib With or Without Ipilimumab for Advanced Hepatocellular Carcinoma: Results From Cohort 6 of the CheckMate 040 Trial. *J Clin Oncol*, **41**, 1747–1757.

Zdrazil,B. *et al.* (2024) The ChEMBL Database in 2023: a drug discovery platform spanning multiple bioactivity data types and time periods. *Nucleic Acids Res*, **52**, D1180–D1192.

Zhang,S.-D. and Gant,T.W. (2008) A simple and robust method for connecting small-molecule drugs using gene-expression signatures. *BMC Bioinformatics*, **9**, 258.

Zhu,A.X. *et al.* (2019) Ramucirumab after sorafenib in patients with advanced hepatocellular carcinoma and increased α-fetoprotein concentrations (REACH-2): a randomised, double-blind, placebo-controlled, phase 3 trial. *Lancet Oncol*, **20**, 282–296.

**10. Glossary**

| Term | Definition |
| --- | --- |
| AUC | **Area Under the Curve** - A metric used to evaluate the performance of signature search method and TopN. |
| CMap | **Connectivity Map** - A project and database that aims to systematically explore the molecular signatures of small molecules, providing a resource for researchers to discover connections between drugs, genes, and diseases. |
| DEG | **Differentially Expressed Genes** - Genes that show a significant change in expression levels under different conditions, such as between disease states or in response to a treatment. |
| ES | **Enrichment Score** - Another metric used to evaluate the performance of signature search method and TopN. |
| FDA | **U.S. Food and Drug Administration** - The national authority of the U.S. is responsible for protecting and promoting public health through the control and supervision of food safety, tobacco products, dietary supplements, prescription and over-the-counter pharmaceutical drugs, and medical devices. |
| FDR | **False Discovery Rate** - A statistical measure used to control the expected proportion of false positives among the rejected hypotheses in a set of tests. |
| GEO | **Gene Expression Omnibus** - A public database of high-throughput gene expression data and other functional genomics experiments, supported by the National Center for Biotechnology Information (NCBI). |
| GSEA | **Gene Set Enrichment Analysis** - A computational method used to determine whether a set of genes shows statistically significant, coherent differences between two biological states or conditions. |
| IC_50_ | The concentration of a drug that inhibits the growth of a cell culture by 50%. It is a common measure of a drug's potency in pharmacology. |
| ICGC | **International Cancer Genome Consortium** - An international collaboration that aims to promote the analysis and sharing of genomic data on human cancers to better understand the genetic basis of cancer. |
| log2FC | **Logarithm base 2 of the fold change** - A common term used to describe the magnitude of change in gene expression levels between different conditions or samples |
| SS_all | **Signature Search All** - A method used in pharmacotranscriptomic analysis that integrates multiple Signature Search Methods (SSMs) to identify drugs with consistent effects across different oncogenic signatures or conditions. It aims to find drugs that are consistently ranked high by various SSMs, indicating a strong potential for repurposing. |
| SS_cross | **Signature Search Cross** - A comparative analysis method that evaluates the response of drugs based on two distinct oncogenic signatures or conditions. It helps to identify drugs that show a similar effect in both signatures, which could indicate a shared mechanism of action or therapeutic potential for multiple disease states. |
| SSM | **Signature Search Method** - An approach used to identify drugs or conditions that produce a similar gene expression signature to a query signature. |
| SSP | **Signature Search Polestar** – Name of website for drug repurposing |
| TCGA | **The Cancer Genome Atlas** - A landmark cancer genomics project that aimed to catalog the genetic changes in various types of cancer and provide this information to the scientific community for further research. |
| XCos | A method for measuring the cosine similarity between gene expression profiles, which can be applied to identify drugs with similar effects to a query gene signature or to compare different gene expression datasets. |
| XSum | A method for evaluating the similarity between gene expression signatures, often used in the context of drug repurposing and disease gene expression analysis. |
| ZhangScore | A scoring method developed for assessing the connectivity between gene sets and small molecules, used in pharmacogenomics to predict drug-target interactions based on gene expression data. |
